# Development and characterisation of a novel 3D in vitro model of obesity-associated breast cancer as a tool for drug testing

**DOI:** 10.1038/s41523-025-00766-3

**Published:** 2025-05-30

**Authors:** Rhianna R. R. Blyth, Stèphanie A. Laversin, Russell B. Foxall, Constantinos Savva, Ellen Copson, Ramsey I. Cutress, Charles N. Birts, Stephen A. Beers

**Affiliations:** 1 Antibody and Vaccine Group, Centre for Cancer Immunology, School of Cancer Sciences, Faculty of Medicine, University of Southampton, Southampton, SO16 6YD UK; 2 School of Cancer Sciences, Faculty of Medicine, University of Southampton, Southampton, SO16 6YD UK; 3 NIHR Southampton Biomedical Research Centre, University Hospital Southampton NHS Foundation Trust, Southampton, SO16 6YD UK; 4 School of Biological Sciences, Faculty of Environmental and Life Sciences, University of Southampton, Southampton, SO17 1BJ UK; 5 Institute for Life Sciences, University of Southampton, Southampton, SO17 1BJ UK

**Keywords:** Breast cancer, Cancer metabolism, Cancer microenvironment, Cancer models

## Abstract

Obesity is associated with worse breast cancer outcomes and decreased therapeutic efficacy. However, the mechanisms driving obesity-associated therapy resistance remain unclear; in part due to a lack of suitable models that recapitulate the obese tumour microenvironment. To address this, we developed a 3D in vitro model of obesity-associated breast cancer, to investigate biological mechanisms and to use as a drug testing tool. A penta-culture system was developed by co-culturing adipocyte spheroids with breast tumour cells, myoepithelial cells, macrophages, and fibroblasts in a collagen matrix. Tumour cells and macrophages infiltrated adipocyte spheroids, replicating the inflamed-adipose border typical of obese patients. This model was then assessed as a drug testing platform. Obese cultures exhibited increased sensitivity to metformin and, conversely, resistance to paclitaxel, compared to non-obese cultures. This 3D organotypic model effectively recapitulates key features of the obese adipose tumour microenvironment, providing a useful tool to interrogate mechanisms underpinning obesity-related therapy resistance.

## Background

Breast cancer (BC) is the second leading cause of cancer-related deaths in women globally^1^. Obesity is known to increase BC risk in postmenopausal women with oestrogen receptor (ER)-positive disease^2^, although the underlying biological mechanisms are still largely unknown. Furthermore, regardless of menopausal status or age, BC patients with obesity have a worse prognosis compared to patients with a healthy BMI^3,4^.

Pre-clinical and clinical studies have reported that obesity-induced changes in the tumour microenvironment (TME) and surrounding breast tissue, promotes BC progression and aggressiveness^5,6^. Adipose tissue (AT) surrounds the mammary gland, and the adipose microenvironment, containing a milieu of cell types; adipocytes, adipose-derived stem cells, fibroblasts, endothelial and immune cells, may play a major role in BC development. For example, adipokines and proinflammatory cytokines secreted from obese AT have been shown to promote tumour progression^7,8^. Metabolic dysregulation and hypertrophy of adipocytes is characteristic of obesity, due to increased lipid storage. The transfer of lipids between AT and tumour cells has recently been proposed as a key process promoting BC progression and aggressiveness^9,10^, although this remains largely underexplored.

Obesity has also been associated with reduced chemotherapy efficacy^11,12^. Decreased response to chemotherapy may be due to changes in the breast TME. Breast tumours from women with obesity feature desmoplasia, characterised by an increased density of cancer-associated fibroblasts (CAFs) and alterations to the extracellular matrix (ECM)^13,14^. Adipocyte de-differentiation, via BC-induced lipolysis, has been suggested to contribute to desmoplasia and generate a pro-tumour microenvironment^15^. Obesity has also been shown to alter the function of immune cell populations within AT, and these changes may also be reflected in the TME, impacting therapy response^16^. Chronic inflammation induced by adipose tissue macrophages (ATMs), and the presence of macrophages surrounding necrotic adipocytes; forming crown-like structures (CLS) is a hallmark of obesity^17–19^.

Increasing efforts have focused on investigating the underlying biology of obesity-associated BC, where studies have centred on mechanisms involved in chronic inflammation, adipokines, and oestrogen signalling. There is, however, a lack of understanding of the link between increased adiposity and BC biology, as most of the molecular evidence comes from pre-clinical animal models. In 1959, Russell and Birch proposed the Three R’s of animal research: Replacement, Refinement and Reduction^20^, laying the foundation for the development of alternatives to the use of animals in research^21^. In accordance with this and the establishment of other organisations such as the National Centre for the Replacement Refinement & Reduction of Animal Models in Research (NC3R’s)^22^, recent efforts have centred on developing 3D in vitro models that better recapitulate the breast TME. As reviewed in^23^, such models typically involve culturing breast epithelial cells alone^24,25^ or with fibroblasts^26–28^ in 3D in the presence of ECM such as Matrigel^TM^ or collagen type I.

However, there remains a paucity of 3D BC models which have incorporated human adipocytes, likely due to the challenges of culturing these cells. Additionally, traditional 2D in vitro cell culture techniques are unsuitable for long term adipocyte differentiation and culture due to their high lipid content, which makes them buoyant, resulting in cell detachment from the cell culture plate^29^. Furthermore, tissue culture plastic is not optimal for adipocyte culture, resulting in decreased adipogenic potential and increased fibroblast-like phenotype when in contact with a plastic surface^29^.

To our knowledge, no validated experimental model exists to investigate the mechanisms of therapeutic resistance associated with the inflammatory adipose environment in BC patients with obesity. Most previous research has utilised transwell culture systems to investigate the impact of adipocytes on breast tumour cells. However, this does not allow for direct cell-cell contact or cell-ECM interactions. Our approach provides a new strategy to mimic caloric overload in a human 3D organotypic penta-culture system, which will support studies to elucidate the mechanisms by which obese adipocytes contribute to BC progression and provide a useful tool for testing therapeutics against BC in a setting that models obesity.

## Results

### Developing a 3D organotypic model of oestrogen receptor-positive breast cancer

**Fig. 1:**
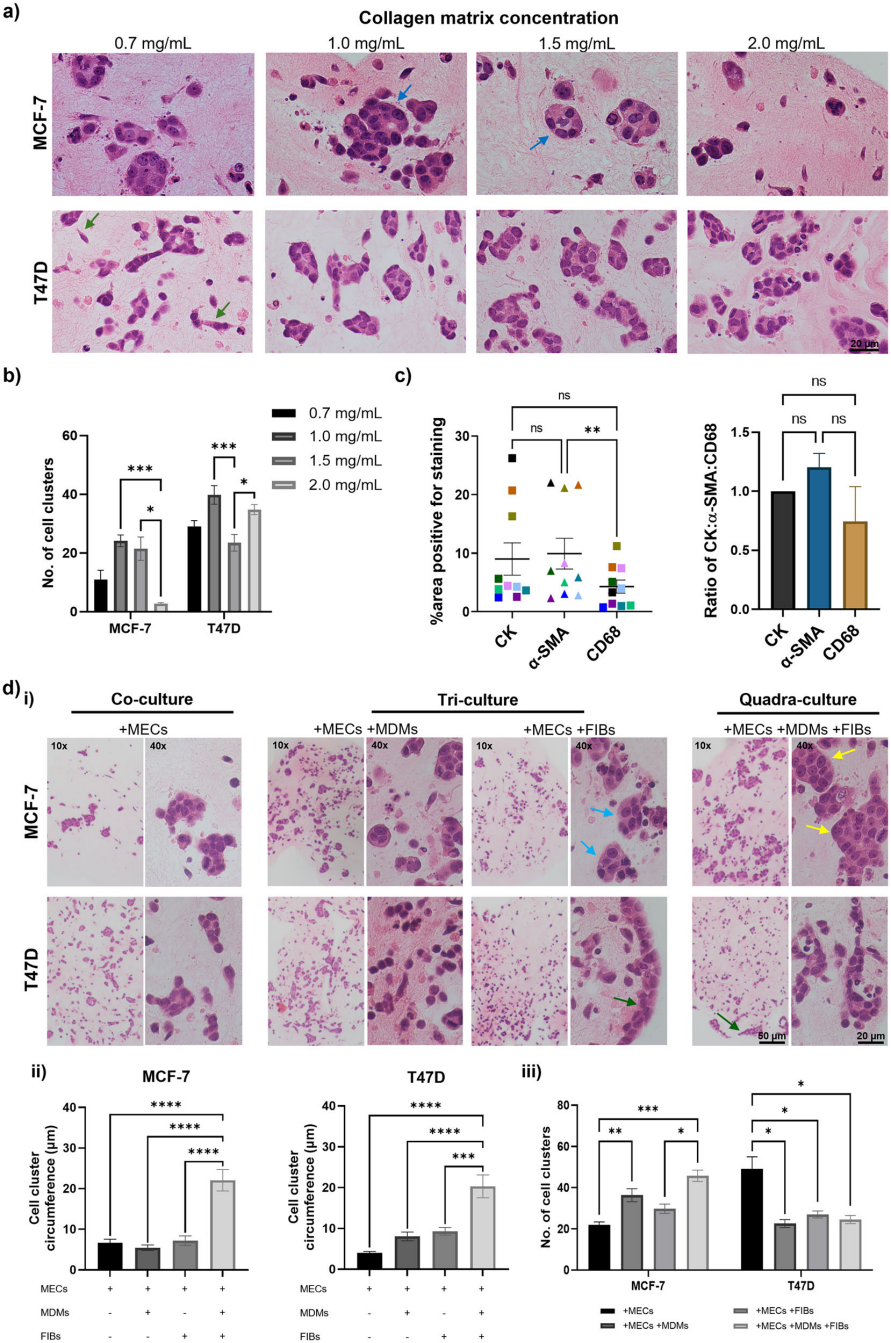
Developing a physiologically relevant organotypic model of ER+ breast cancer. **a** MCF-7 and T47D breast tumour cells were cultured in 3D in collagen type I matrices ranging in concentration from 0.7–2.0 mg/mL for 7 days. H&E stained 4 µm sections of Formalin-fixed Paraffin-embedded (FFPE) organotypic cultures are shown. Each brightfield image is obtained from a single organotypic model at 40x magnification, scale bar: 20 µm. Arrows indicate morphologies of interest; either mass (blue) or mesenchymal (green). **b** Number of cell clusters in different concentrations of collagen matrix. Cell clusters counted in ImageJ from three images per treatment, across three experiments (*n* = 9). **c** Percentage positive staining of pan-cytokeratin (filled squares), α-SMA (filled triangles) and CD68 (filled squares) from images of multiplex IHC slides from full face sections of ER+ and TNBC breast cancer samples from the BeGIN study (Breast Cancer: Correlating Genetic, Immunological and Nutritional Predictors patient cohort; not published), colour coded according to patient ID. **d** Breast tumour cells (MCF-7 or T47D) co-cultured with myoepithelial cells (MECs) in a type I collagen matrix for 7 days. Tri-cultures contained monocyte-derived macrophages (MDM) or HFFF2 fibroblasts (FIBs), and quadra-cultures contained all four cell populations. (i) Arrows highlight morphologies of interest; mass clusters (blue and yellow) and tumour islands at the collagen edge (green). Images taken at 10x and 40x magnification under brightfield. Scale bars: 50 and 20 µm, respectively. (ii) Circumference of cell clusters in each culture condition. (iii) Number of cell clusters with 4 or more nuclei in each field of view. Quantification performed on three fields of view from *n* = 3 organotypic samples. Data represent mean ± SEM with **c** one-way ANOVA with Friedman test or **d** two-way ANOVA used to test for statistical significance; *P < 0.05, **P < 0.01, ***P < 0.001, ****P < 0.0001 and non-significant (ns) *P* > 0.05.

Previous reports support a greater effect of BMI on luminal breast cancer risk than non-luminal subtypes^30,31^. Therefore, we set out to develop a novel 3D in vitro model of luminal A disease using two well-characterised cell lines. We selected type I collagen to recapitulate the ECM of primary breast tumours, as this is the major constituent of breast stroma^32^. As previous studies have shown that breast tumour cells of different subtypes exhibit optimal growth when cultured in matrices of varying stiffness^33–35^, we first determined the collagen concentration required for the 3D culture of two ER^+^ breast tumour cell lines: MCF-7 and T47D. Each breast tumour line was first seeded into a collagen type I matrix of varying concentrations, ranging from 0.7 to 2.0 mg/mL and cultured for seven days. 3D culture of MCF-7 cells in collagen resulted in the formation of solid nests and gland-like structures, exhibiting filled lumens (Fig. 1a). In accordance with an earlier study, MCF-7 cells formed structures classified as showing a ‘mass’ morphology, where the nucleus is disorganised, and cell-cell adhesion is strong (Fig. 1a, blue arrows)^24^. MCF-7 cells formed the greatest number and size of cell clusters in a 1.0 mg/mL collagen matrix (Fig. 1a, b). T47D cells also developed tumour nests when cultured in a 3D matrix, also exhibiting a ‘mass’ morphology (Fig. 1a, bottom row). Although the greatest number of T47D gland-like structures was observed in a matrix of 1.0 mg/mL (Fig. 1b), their morphology was similar across all collagen concentrations tested. Interestingly, a lower collagen concentration of 0.7 mg/mL resulted in T47D cells adopting a mesenchymal morphology (Fig. 1a, green arrows). In comparison, in 3D culture of the TNBC cell line MDA-MB-231, cells did not allow for gland-like structure formation, with this cell line maintaining a mesenchymal morphology (Fig. S1a), characteristic of its invasive phenotype^36^. Notably, at a higher collagen concentration of 1.5 mg/mL, MDA-MB-231 cells showed increased cellular interactions and the formation of large cell clusters (Fig. S1a), whereas, at 2.0 mg/mL, cell clusters only formed around the outer edges of the collagen gel (Fig. S1a). Therefore, a collagen concentration of 1.0 mg/mL was selected for MCF-7 and T47D 3D cell cultures.

In order to develop a novel in vitro model which reflects the breast TME, we initially established a quadra-culture system, based on a previous study^26^, consisting of breast tumour cells, myoepithelial cells, fibroblasts and monocyte-derived macrophages. To maximise the physiological relevance of co-cultures, we analysed multiplex IHC staining of punch biopsies from primary breast cancers of either ER^+^ or TN disease subtypes to determine the ratio of breast cancer cells to fibroblasts and macrophages. This was determined by assessing the percentage area positive for pan-cytokeratin (CK), a pan-breast cancer cell marker, α-SMA; a myofibroblast marker, and CD68 as a pan-macrophage marker. Across the 10 samples analysed, there was no significant difference between the areas of cells positive for CK and α-SMA (*P* = 0.2858) in ER^+^ cases (Fig. 1c). While not statistically significant, a trend towards fewer CD68+ cells compared to CK+ cells was observed across the independent experiments. Therefore, a ratio of 1:1:0.75 was selected for breast tumour cells: fibroblasts: macrophages for our model system (Fig. 1c). Of note, in TNBC cases, there were significantly more α-SMA+ cells than tumour cells, with a ratio of 2.5:1 fibroblasts: breast tumour cells (Fig. S1b). Myoepithelial cells (MECs) are often lost or decreased in number during breast cancer progression, and this is particularly evident in obesity^37^. Therefore, a ratio of 1:0.5 breast tumour cells: MECs was used for all co-culture systems. A growing number of studies have shown that tumour-associated MECs have a tumour-promoting effect^38–40^. Furthermore, the transition of MECs from possessing a tumour suppressive nature in DCIS to adopting a tumour supporting role in invasive carcinomas has been previously reported in an obese setting^41^. Therefore, although MECs are often thought of as unnecessary in modelling invasive disease, we have elected to incorporate MECs into our organotypic system to investigate their role in BC progression in an obese setting. Our results showed that MCF-7 breast cancer cells underwent morphological changes when co-cultured with other cell populations of the TME (Fig. 1d). Tri-culture of MCF-7 or T47D cells cultured with MECs and monocyte-derived macrophages (MDMs) in a 1.0 mg/mL collagen matrix displayed solid tumour nests (Fig. 1di). Whereas tri-cultures with fibroblasts (FIBs) and MECs resulted in nests that were irregular in shape (Fig. 1di, blue arrows). In comparison, quadra-cultures containing MCF-7 cells generated large irregular tumour nests (Fig. 1di, yellow arrows and 1d-ii), suggestive of increased tumour cell proliferation. In the presence of fibroblasts, T47D cells formed larger clusters (Fig. 1dii), mainly around the edge of the collagen gel (Fig. 1d, green arrows). Irrespective of co-culture conditions, T47D cells formed fewer tumour nests that were spread throughout the matrix compared to monocultures (Fig. 1diii).

### Generating hypertrophic adipocytes to model obesity

**Fig. 2:**
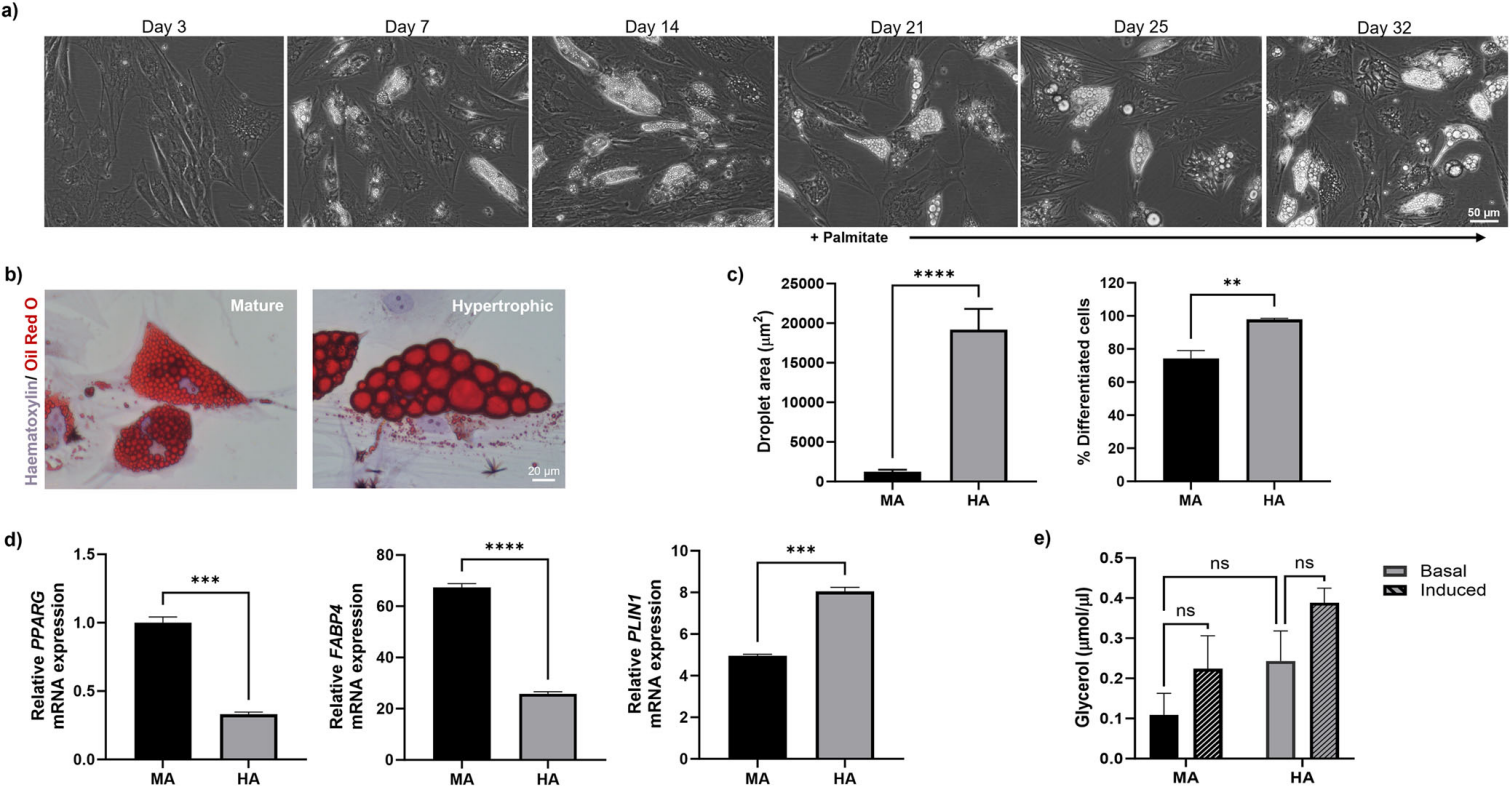
Palmitate induces a hypertrophic-like phenotype in adipocytes. Adipose-derived stem cells accumulate cytoplasmic lipid droplets seven days post-differentiation. Mature adipocytes at day 21 contain small lipid droplets. After supplementation with 350 µM palmitate, adipocytes adopt a hypertrophic-like phenotype, exhibiting larger lipid droplets. **a** Representative phase contrast images of ADSC undergoing adipocyte differentiation, scale bar indicates 50 µm. **b** Representative brightfield images depicting Oil Red O staining of lipid droplets in mature (left-hand image) and hypertrophic (right-hand image) adipocytes. Scale bar indicates 20 µm. **c** The area of lipid droplets and percentage of differentiated cells was measured in ImageJ. Data was obtained by scoring three fields of view at 40x magnification from three wells of a 6-well plate across three experiments. **d** Relative mRNA expression of PPARG, FAPB4 and PLIN-1 in 2D culture of adipocytes compared to β-actin mRNA expression. **e** Amount of glycerol released after 3 h in the presence (induced) or absence (basal) of isoprenaline at the culture endpoint of mature and hypertrophic adipocytes. All data reported as mean ± SEM of *n* = 3 independent experiments. Statistical significance between groups was assessed using unpaired *t*-tests, ****P < 0.0001, ***P < 0.001, **P < 0.01 and ns non-significant *P* > 0.05.

To establish a physiologically relevant model of obesity-associated BC, adipocytes were incorporated to develop a penta-culture system. Firstly, ‘mature’ adipocytes (MA) were differentiated from human adipose-derived stem cells (ADSCs) by culturing in adipogenic media for 21 days. ADSCs began to display morphological characteristics of adipocytes from day 7 of differentiation, accumulating cytoplasmic lipid droplets, which increased in size over the 21-day culture period (Fig. 2a). To better replicate the obese adipose microenvironment, hypertrophic-like adipocytes (HA) were generated via palmitate treatment of MA, a process reported to mimic caloric overload^42,43^. This treatment resulted in a significant increase in lipid droplet size and percentage of cells successfully differentiated in HA compared to (Fig. 2b, c).

Real-time metabolic analysis measuring glycolysis, oxidative phosphorylation (OXPHOS) and fatty acid oxidation (FAO) was used to assess the metabolic profile of MA and HA. Overall, as expected given the slow proliferation rate associated with maturation in vitro^44^, both MA and HA featured low metabolic activity (Fig. S2). Extracellular acidification rate (ECAR) is indicative of glycolytic lactate production. At baseline, in an environment starved of glucose, total ECAR was similar in both MA and HA (Fig. S2a). Addition of glucose led to a more pronounced rise in basal ECAR in HA compared to MA (Fig. S2a), however, they showed similar levels of glycolytic capacity. This may indicate that under basal conditions, both are working at maximal glycolytic rate (Fig. S2b). However, HA had a significantly reduced glycolytic reserve compared to MA (*P* < 0.01) (Fig. S2b).

Oxygen consumption rate (OCR) is indicative of OXPHOS. Basal OCR was low in HA and MA compared to the maximal OCR achieved through FCCP, suggesting that these adipocytes are not heavily reliant on OXPHOS (Fig. S2c, d). However, HA did feature a trend toward increased mitochondrial OCR compared to their mature counterparts.

Treatment of adipocytes with Etomoxir, an inhibitor of long-chain fatty acid transport into the mitochondria, reduced OCR levels and spare respiratory capacity in both HA and MA (Fig. S2e). However, HA showed a trend towards increased FAO compared to MA (Fig. S2f). Therefore, this may support that long-chain fatty acids are being utilised for energy production via OXPHOS in HA. Overall, we observed a trend that HA showed greater maximal respiration and spare respiratory capacity and that this may be fuelled by increased FAO.

As the metabolic profiles of MA and HA were largely similar (Fig. S2), we sought to assess three key adipogenic markers at the transcript level to determine whether this method for generating HA produced metabolically dysregulated adipocytes typical of obesity. In 2D culture, HA exhibited a significant decrease in expression levels of *PPARG* and *FABP4* as compared to MA (Fig. 2d). Conversely, mRNA expression levels of *PLIN-1* were increased in HA compared to MA. We also observed a trend towards higher basal lipolysis for HA compared to MA when cultured in 2D (Fig. 2e). Of note, isoprenaline treatment did not further induce lipolysis in HA, which is characteristic of adipocyte hypertrophy^45^. However, isoprenaline treatment of MA also only showed a trend towards increased lipolysis activity (Fig. 2e).

**Fig. 3:**
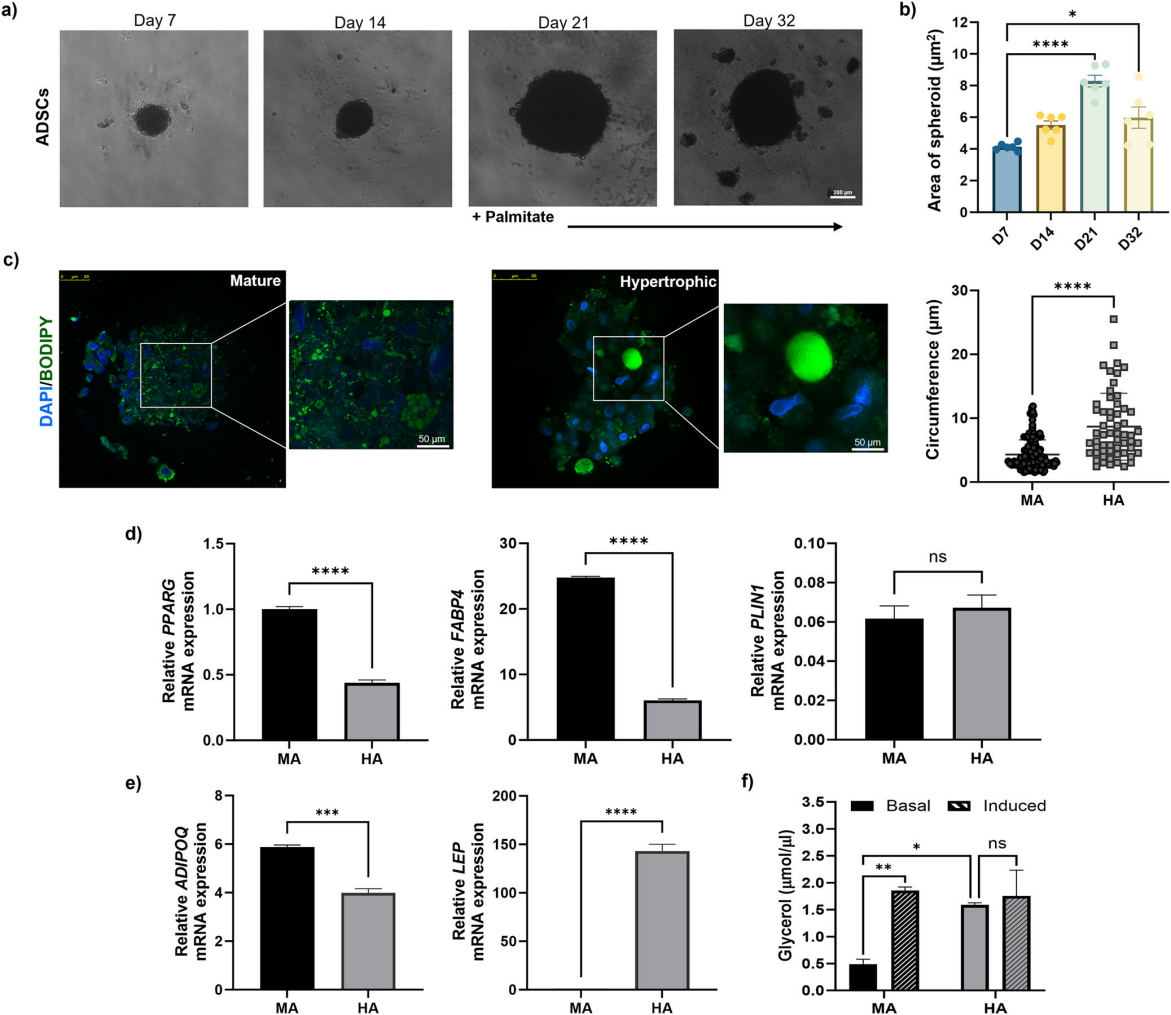
Hypertrophic-like adipocyte spheroids have larger lipid droplets and exhibit metabolic dysregulation. **a** Brightfield images of adipose-derived stem cells (ADSCs) seeded in ultra-low attachment plates and differentiated over a period of 32 days. Representative phase contrast images of spheroids; 4x magnification, scale bar represents 200 µm. **b** The area of spheroids measured in ImageJ. Data represent the mean ± SEM of *n* = 6 spheroids. Statistical significance between groups was assessed using one-way ANOVA tests. **c** Confocal IF images of fixed MA (day 21) or HA (day 32) spheroids, co-stained with BODIPY and DAPI; 20x magnification, scale bar represents 50 µm. The graph shows the mean circumference of BODIPY-positive MA (black) and HA (grey) spheroids. Values were generated using ImageJ, average of *n* = 3 fields of view. **d** Relative mRNA expression of PPARG, FAPB4 and PLIN-1 in 3D culture of adipocytes compared to β-actin mRNA expression. **e** Relative mRNA expression of ADIPOQ and LEP in 3D culture of adipocytes compared to β-actin mRNA expression. **f** Amount of glycerol released after 3 h in the presence (induced) or absence (basal) of isoprenaline at the culture endpoint of MA and HA in 3D spheroid culture. All data reported as mean ± SEM across *n* = 3 independent experiments. ****P < 0.0001, **P < 0.01, *P < 0.05, ns *P* > 0.05.

Incorporating adipocytes into organotypic systems is challenging due to their high lipid content and rapid de-differentiation. To overcome this obstacle, adipocytes were cultured as spheroids. ADSCs were cultured in 2D until 70% confluent, and then seeded into ultra-low attachment plates. ADSCs were then differentiated in 3D as outlined above for 21 days until classed as mature and then treated with palmitate to induce a hypertrophic phenotype (Fig. 3a). This method generated viable spheroids, that increased in circumference over a period of 21 days (Fig. 3b). However, addition of palmitate to adipocyte spheroids did result in a degree of disruption and consequent reduction in overall spheroid size (Fig. 3a, b). ADSCs were successfully differentiated in 3D into adipocytes after 21 days of culture. As seen in Fig. 3c, spheroids have formed adipose-like structures: large spherical cells, containing unilocular lipid droplets. Following palmitate treatment, these cells significantly increased in size relative to untreated MA (Fig. 3c).

In agreement with our 2D culture data (Fig. 2d), HA cultured as 3D spheroids showed significantly decreased mRNA expression levels of *PPARG* and *FABP4* compared to MA (Fig. 3d). However, mRNA expression levels of *PLIN1* were similar in HA and MA when cultured in 3D. This differed from 2D culture, where HA showed increased mRNA expression levels of *PLIN1* compared to MA. Spheroid culture of HA also displayed decreased *ADIPOQ* and increased *LEP* mRNA expression levels compared to MA (Fig. 3e), indicative of an obese phenotype^46^. Additionally, HA spheroids exhibited a significant increase in lipolysis compared to MA and isoprenaline treatment was unable to induce further lipolysis in HA (Fig. 2f). Comparatively, induced lipolysis of MA spheroids upon treatment with isoprenaline significantly increased the amount of glycerol released into the culture media (Fig. 2f), which was not observed in 2D culture. In addition to this, adipocyte spheroids also displayed differential metabolic profiles. A significant increase of basal glycolysis (Fig. S3a, b), and basal mitochondrial respiration (Fig. S3c, d) was observed in HA spheroids compared to their mature counterparts. Overall, the downregulation of MA markers in HA and enhanced lipolytic activity, whether cultured in 2D or 3D, supported that they have adopted a metabolically dysregulated phenotype. As HA spheroids showed significantly enhanced lipolytic activity compared to MA, which was only an observed trend in 2D culture, this may suggest that 3D spheroid culture of adipocytes are a more suitable method of generating HA.

### Impact of adipocytes on breast tumour cell morphology and cellular localisation

**Fig. 4:**
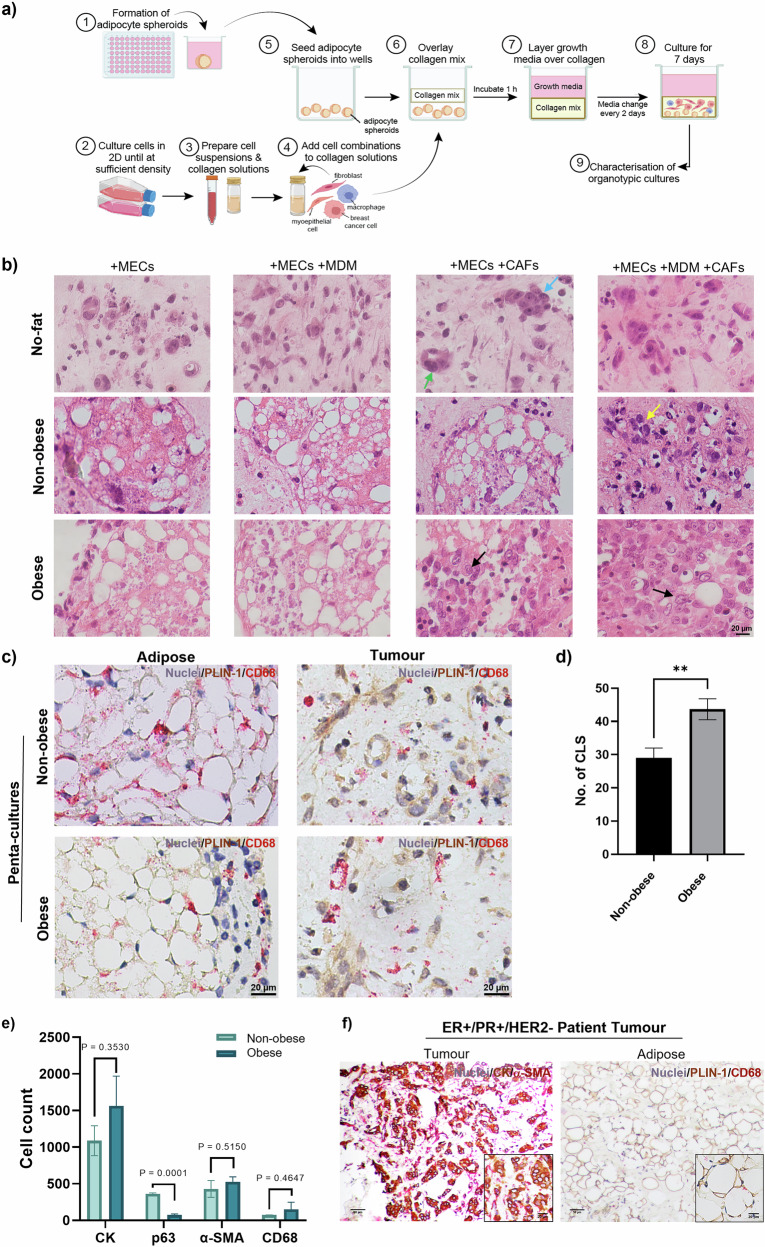
Development of an obese organotypic model of breast cancer using adipocyte spheroids. **a** Schematic overview of generating organotypic cultures. Adipocyte spheroids were generated using ultra-low attachment plates. Cell suspensions were prepared at the required cell density and added to a 1.0 mg/mL collagen type I matrix. Adipocyte spheroids were seeded into the bottom of a 96-well plate, and the collagen-cell solution was gently overlayed. The collagen was then incubated at 37 °C to polymerise before overlaying growth medium. Organotypics were cultured for 7 days and then characterised. Schematic created in BioRender. **b** H&E staining of organotypic co-culture samples. MCF-7 breast tumour cells co-cultured with primary myoepithelial cells (MECs) alone or in combination with monocyte-derived macrophages (MDMs), and cancer-associated fibroblasts (CAFs) in a collagen type I matrix for 7 days in the absence of adipocyte (no-fat), or presence of MA (non-obese) or HA (obese). Scale bar = 20 µm. Blue and green arrows indicate mass-like and gland-like morphology of tumour cells, respectively. Yellow arrow indicates tumour cell migration into the adipocyte spheroid. **c** MCF-7 penta-cultures containing MA or HA spheroids in a 1.0 mg/mL collagen matrix. Organotypic sections stained for perilipin (PLIN-1-brown) and CD68 (Refine red). Haematoxylin is used to counterstain nuclei. Representative brightfield images taken at 40x magnification are shown. Scale bar = 20 µm. **d** Number of crown-like structures across whole penta-culture sections, scored as CD68^+^ cells encapsulating >50% of adipocytes. **e** Average counts of cells positive for CK, p63, α-SMA or CD68 in organotypic samples. Three fields of view from *n* = 3 organotypics per condition were scored. Images were converted into composites using the Colour Deconvolution package in ImageJ and then manually counted for cells positive for each marker. Data represents the mean ± SEM. Statistical significance between groups was assessed using unpaired *t*-tests. **f** Representative images of a human tissue section from a luminal breast cancer patient, stained with CK (brown), α-SMA (red) in the tumour region (left image), PLIN-1 (brown) and CD68 (red) in the adipose region. Scale bar indicates 50 or 20 µm as indicated.

As a next step, we embedded adipocyte spheroids into organotypic cultures (see schematic in Fig. 4a). As above, these were cultured for 7 days prior to phenotypic and functional characterisation.

In a no-fat model (absence of adipocytes), breast tumour cells displayed a ‘mass’ morphology, characterised by tumour nests with irregular nuclei and filled lumen (Fig. 4b- blue arrow). In comparison, in cultures containing MA; where tumour cells localised around adipocytes, there was a higher proportion of tumour cells that developed a gland-like morphology (Fig. 4b- green arrow). However, breast tumour cells dispersed throughout the collagen matrix exhibited both gland-like and mass-like morphologies (Fig. S4a). We noted tumour cell infiltration into MA spheroids, which increased in the presence of primary cancer-associated fibroblasts (CAFs) (Fig. 4b, yellow arrow). Most notably, in the presence of CAFs, there was increased infiltration of breast tumour cells and macrophages into and surrounding the adipocyte spheroids (Fig. 4b, black arrows), recreating the inflamed adipose-tumour border observed in obese breast cancer patients^47,48^.

To investigate cellular localisation and interactions in our organotypic penta-culture system, immunohistochemical staining was performed on organotypic samples. To identify crown-like structures (CLS) in penta-cultures, organotypics were stained for PLIN-1 and CD68. We observed the formation of CLS in both non-obese and obese settings (Fig. 4c). Obese penta-cultures showed a significant increase in the number of CLS compared to non-obese cultures (Fig. 4d), consistent with previous studies^19,49^. Additionally, within the tumour region, tumour cells were positive for PLIN-1, which could represent the transfer of lipids from adipocytes to tumour cells (Fig. 4c, right images). The Colour Deconvolution plugin in ImageJ was used to create composites of original brightfield images, to determine the number of cells positive for specific markers. Using p63 as an MEC marker, we identified the presence of MECs surrounding breast tumour cells (Fig. S4b). Interestingly, in penta-cultures containing HA, there was a significant reduction in p63-positive cells (Fig. 4e), a phenomenon reported in previous studies^37,41^. Of note, in this model system, the presence of HA resulted in a shift in morphology of BC cells from gland-like structures to more invasive sheets of tumour cells spread throughout the collagen matrix compared to cultures with MA (Fig. 4c and Fig. S4b). We also noted a trend towards an increase in pan-cytokeratin positive breast tumour cells in an obese compared to a non-obese setting, which may suggest increased proliferation of breast tumour cells (Fig. 4e).

Myofibroblasts, identified as α-SMA-positive cells, were mainly localised around tumour cells in penta-cultures containing MA (Fig. S4b, blue arrows). However, in an obese setting, they appeared to be more dispersed throughout the collagen matrix. There was also a trend towards increased fibroblast density in obese compared to non-obese organotypics, although this did not reach significance (Fig. 4e). Similarly, macrophages were largely spread throughout the collagen matrix in the presence of MA. Whereas, in cultures containing HA, macrophages were mainly localised around areas rich in breast tumour cells (Fig. S4b, yellow arrows). Our non-obese model recapitulated the morphology of tumour cells observed in lean patients with luminal disease (Fig. 4d and Fig. S4b). Furthermore, the adipocyte spheroids maintained a similar morphology to adipose in the breast (Fig. 4f).

Of note, quadra-cultures showed similar proliferation compared to monocultures for both MCF-7 and T47D (Fig. S4c, d). In comparison, enhanced proliferation was observed in non-obese penta-cultures compared to mono- and quadra-cultures, and this was further increased in an obese setting (Fig. S4c, d).

### Utilising this novel organotypic system as a drug testing platform

**Fig. 5:**
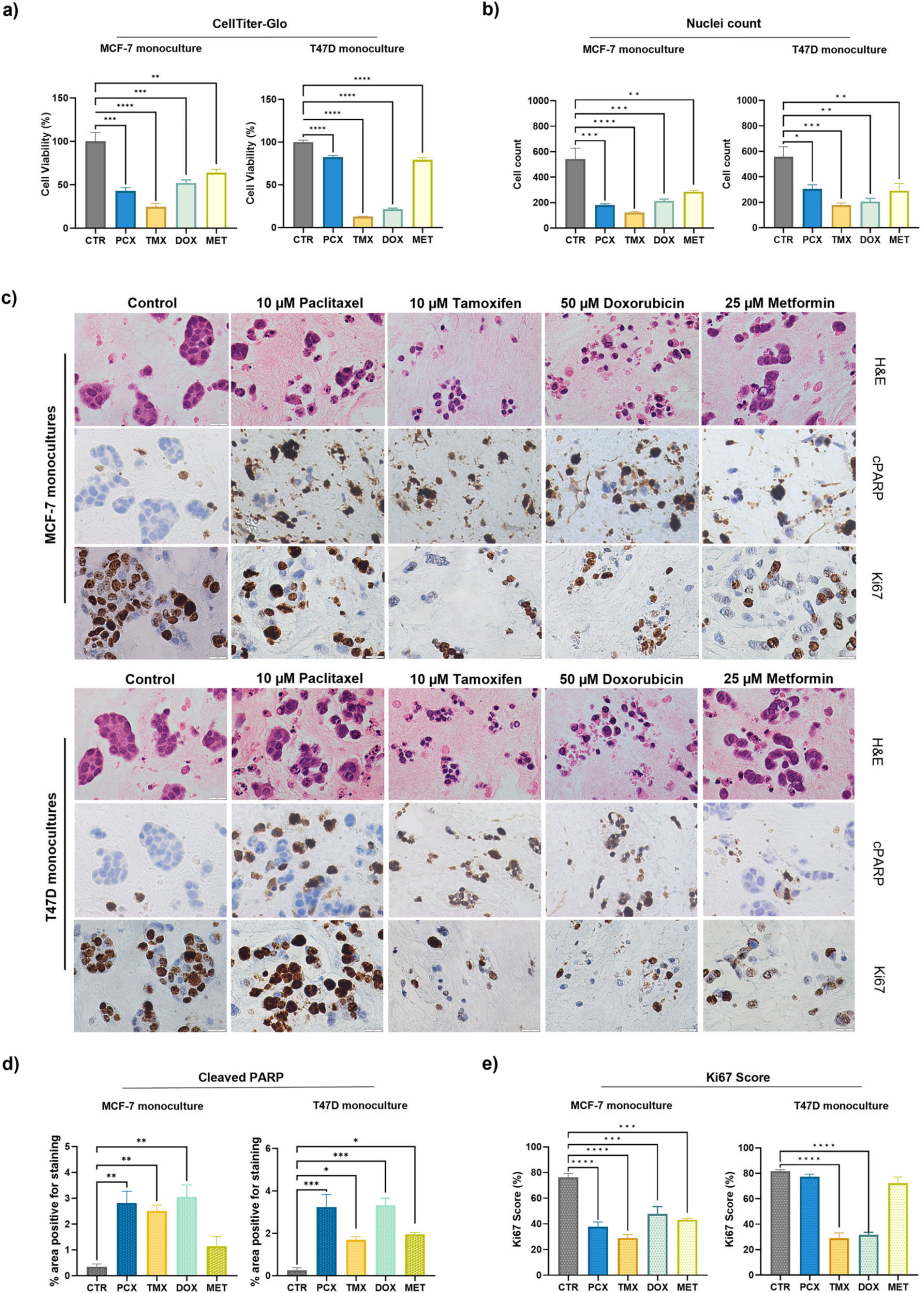
ER+ breast tumour cells are sensitive to paclitaxel and tamoxifen when cultured in 3D. **a** Cell viability was determined with a CellTiter-Glo® 3D assay, according to the manufacturer’s instructions, in breast tumour organotypic monocultures. These cultures were treated for 48 h with one of four therapies: 10 µM paclitaxel, 10 µM tamoxifen, 50 µM doxorubicin, 25 µM metformin, or a DMSO control. Cell viability was determined by converting luminescence values into cell viability by normalising drug-treated cultures to the control cultures. Data were obtained from three independent experiments, with triplicate organotypic samples per treatment condition being scored per experiment. **b**–**e** FFPE blocks generated from treated and untreated organotypic samples were sectioned and stained for H&E, cleaved PARP or Ki67. Representative brightfield images at 40x magnification are shown, scale bar = 20 µm. **b** Number of nuclei manually counted in ImageJ. Sections stained for **d** cleaved PARP (cPARP) or **e** Ki67 were analysed in ImageJ for percentage of area positive for staining using the Colour Deconvolution plugin. Graphs represent the mean ± SEM of *n* = 3 fields of view from *n* = 3 samples per condition. Statistical significance between groups was assessed using an ordinary one-way ANOVA with Dunnett’s or Kruskal–Wallis comparison test. ****P < 0.0001, ***P < 0.001, **P < 0.01, *P < 0.05. All other comparisons were non-significant (*P* > 0.05).

Next, we sought to investigate the potential utility of our model penta-culture system as a drug testing platform. Initially, we tested 3D monocultures of MCF-7 cells incorporated into a collagen matrix to assess drug efficacy and determine the concentrations to use for further experiments in penta-cultures. MCF-7 monocultures were cultured for 7 days and then treated with three different doses of paclitaxel, tamoxifen, doxorubicin or metformin for a further 48 h. These therapeutic agents were selected as they are often used to treat patients with ER^+^ tumours. Endocrine therapy is currently the main therapeutic approach for ER^+^ disease, including anti-oestrogenic compounds, such as Tamoxifen and Fulvestrant. Chemotherapy regimens containing an anthracycline (doxorubicin or epirubicin) and a taxane (paclitaxel or docetaxel) are recommended in national guidance for the treatment of early BC^50^. Therefore, tamoxifen, doxorubicin and paclitaxel were selected as they have been used in similar 3D culture systems^51,52^. Metformin was also selected as recent studies have shown promising results in vitro and in vivo, particularly in the context of obesity^53,54^. Dose ranges were determined from previous studies in 3D BC cultures^51,52^. After 48 h, cell viability assays were performed. Based on the results in Fig. S5, we selected the dosages at which there was approximately a 50% reduction in cell viability. This was then repeated in T47D cells, where 3D monocultures were treated with 10 µM paclitaxel, 10 µM tamoxifen, 50 µM doxorubicin or 25 µM metformin for 48 h. After 48 h, cell viability assays were performed and validated by H&E, cleaved PARP and Ki67 immunohistochemical staining of cultures. MCF-7 3D monocultures were highly sensitive to tamoxifen and paclitaxel, showing a 70% and 50% reduction of cell viability, respectively, compared to untreated controls (Fig. 5a, left-hand panel). Of note, doxorubicin and metformin treatment both reduced cell viability to ~40% of untreated controls (Fig. 5a). T47D cells showed a similar drug response profile, although they were more sensitive to doxorubicin than paclitaxel as compared to MCF-7 cells (Fig. 5a, right-hand panel). This may be due to the higher in vitro proliferation rate of T47D cells and/or the mechanism of action of these two chemotherapeutic agents^55^. Cell viability assays were confirmed by immunohistochemical staining of organotypic sections. To validate the cell viability assays, H&E staining of organotypic samples was performed to quantify the number of cells (Fig. 5b) and assess morphology (Fig. 5c). To further validate the cell viability assays, cleaved PARP staining was performed on MCF-7 and T47D monocultures, to quantify cells undergoing apoptosis (Fig. 5c, d). Ki67 staining was also performed as a proxy of cell proliferation (Fig. 5c, e).

**Fig. 6:**
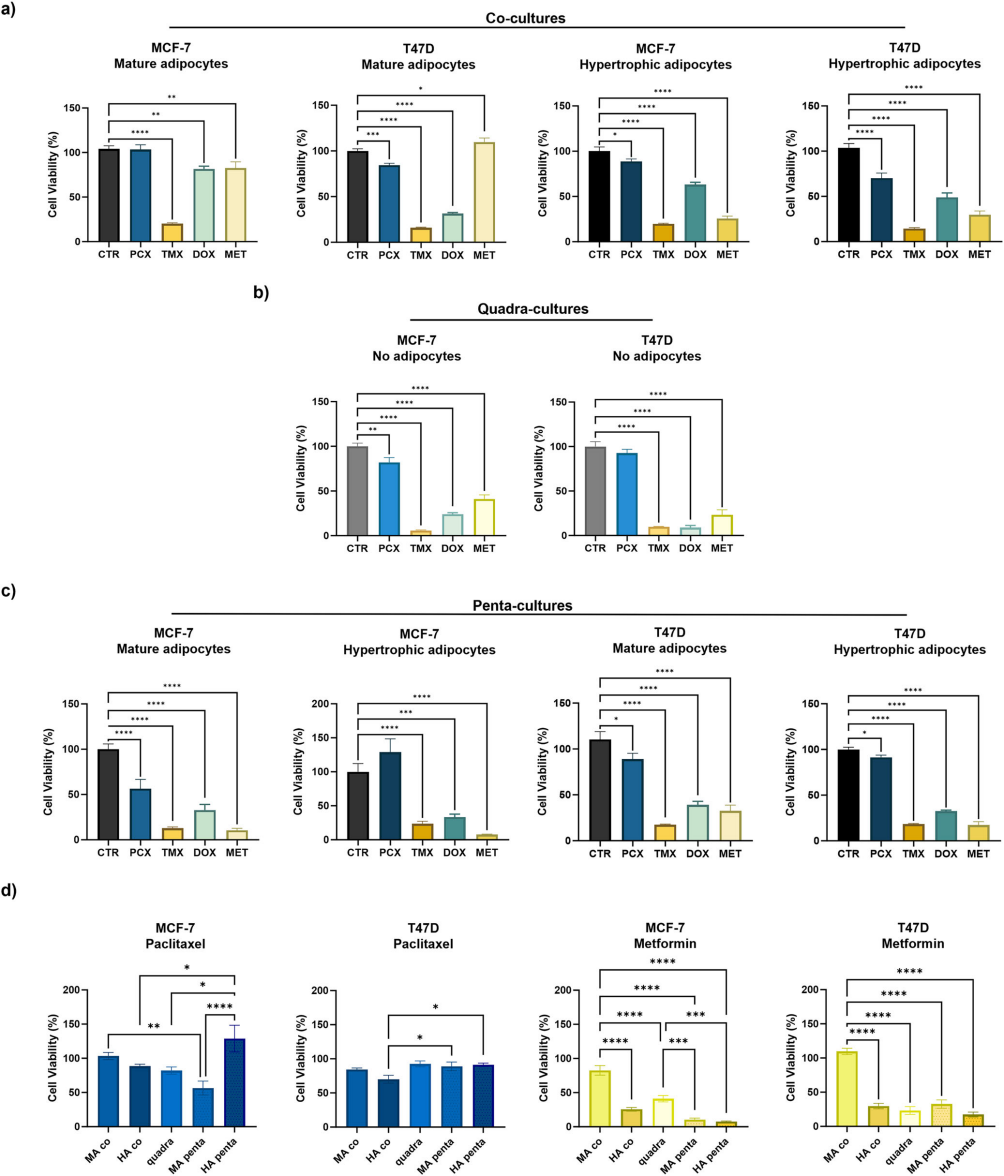
Breast tumour cells are resistant to paclitaxel but sensitive to metformin in an obese setting. Organotypic cultures were treated for 48 h with one of four therapeutic compounds: 10 µM paclitaxel (PCX), 10 µM tamoxifen (TMX), 50 µM doxorubicin (DOX), 25 µM metformin (MET), or a DMSO control (CTR). Cell viability was determined via CellTiter-Glo® 3D assay, according to manufacturer’s instructions, in breast tumour organotypic **a** co-, **b** quadra- or **c** penta-cultures containing either MA or HA. Cell viability was determined by converting luminescence values into cell viability by normalising drug-treated cultures to the control cultures. **d** Summary of paclitaxel and metformin treatment in MCF-7 or T47D organotypics across different co-culture conditions. Data were obtained from three independent experiments, with triplicate organotypic samples per treatment condition. Statistical significance between groups was assessed using an ordinary One-Way ANOVA with Dunnett’s or Kruskal–Wallis comparison test. ****P < 0.0001, ***P < 0.001, **P < 0.01, *P < 0.05. All other comparisons were non-significant (*P* > 0.05).

We next sought to determine the effect of adipocytes on drug sensitivity using a 3D co-culture system of breast tumour cells co-cultured with MA or HA spheroids. Co-culture of either MCF-7 or T47D cells with MA spheroids resulted in paclitaxel resistance and reduced sensitivity to doxorubicin (Fig. 6a). T47D cells co-cultured with HA also exhibited reduced sensitivity to doxorubicin treatment (Fig. 6a). Of note, BC co-cultures with MA were not sensitive to metformin treatment and displayed between 70% and 100% viability for MCF-7 and T47D cells, respectively. In comparison, the presence of HA spheroids restored sensitivity to metformin treatment, where both BC cell lines exhibited around 30% cell viability (Fig. 6a). This suggests that adipocytes may play a role in obesity-associated therapy resistance and may highlight metformin as a potential therapeutic approach for obese breast cancer patients, which has been previously suggested^56^.

To investigate the effects of the breast TME in a no-fat setting, a quadra-culture system was then utilised as a drug testing platform. Breast tumour cells were cultured with MECs, MDMs, and fibroblasts for 7 days and then treated with tamoxifen, doxorubicin, paclitaxel or metformin as above for 48 h. MCF-7 and T47D quadra-cultures showed a similar response to treatments. We observed a decreased sensitivity to paclitaxel, only achieving a reduction in cell viability of ~18 and 7% in MCF-7 and T47D cells, respectively (Fig. 6b). Of note, quadra-cultures were sensitive to doxorubicin and metformin for both BC cell lines (Fig. 6b).

To determine whether fibroblasts and/or macrophages impact obesity-associated resistance to chemotherapy and metformin sensitivity, we repeated the drug sensitivity assays in the context of our novel penta-culture system. In a non-obese setting, penta-cultures containing MA displayed sensitivity to tamoxifen and metformin treatment (Fig. 6c). This was confirmed by immunohistochemical analysis, which revealed a significant decrease in the number of cell nuclei, and increased Caspase-3 staining (Fig. S6a, bii), confirming a significant increase in apoptosis in tamoxifen-treated samples compared to untreated controls. These cultures also exhibited sensitivity to doxorubicin, with an average decrease in cell viability of ~60% (Fig. 6c). Interestingly, Caspase-3-positive cells were mainly localised around adipocytes in doxorubicin-treated penta-cultures (Fig. S6a).

In an obese setting, HA penta-cultures featured high sensitivity to metformin treatment (Fig. 6c). Interestingly, HA penta-cultures maintained sensitivity to doxorubicin, and as in non-obese cultures, Caspase-3-positive cells were mainly localised around adipocytes (Fig. S6a). Most notably, penta-cultures containing HA displayed therapy resistance to paclitaxel (Fig. 6c, dark coloured bars), with cell viability being similar to or greater than the untreated control cultures. When comparing across the culture conditions, it was observed that in an obese setting, MCF-7 penta-cultures developed paclitaxel resistance compared to quadra- or non-obese penta-cultures (Fig. 6d). Additionally, metformin sensitivity was greatest in non-obese and obese MCF-7 penta-cultures compared to quadra-cultures or co-cultures with adipocytes (Fig. 6d). Overall, our data suggests that HA, upon interactions with fibroblasts and macrophages within the obese breast TME, may contribute to chemotherapy resistance in ER+ breast cancer. Furthermore, our data suggests that metformin may provide therapeutic benefit in breast cancer patients with obesity.

## Discussion

Recent studies have highlighted the importance of the adipose microenvironment in BC progression and acquired therapy resistance^12,57,58^. Over the past few decades, various in vitro models of BC have emerged, in an attempt to model the complex interactions of the breast TME. Such models include 2D transwell co-cultures, multicellular spheroids (with and without scaffolds), scaffold-based cell-encapsulated bioprinted/engineered matrices, and ex vivo cultures. As previously reviewed in ref. ^23^, each model varies in its complexity and associated advantages and disadvantages. However, only a limited number of models allow for the study of the impact of the adipose TME on therapeutic response and disease progression^59^.

Therefore, there is a clear need for robust 3D in vitro models incorporating components of the adipose microenvironment. Here, we present a 3D in vitro organotypic model of human breast cancer associated with obesity-induced adipose inflammation. This model incorporates breast luminal epithelium, myoepithelial cells, macrophages, fibroblasts, and adipocytes; embedded in a physiologically relevant matrix of type I collagen. This model reflects the morphology and phenotype of BC in an obese setting, providing a novel tool to investigate the processes supporting the crosstalk between breast tumour cells and adipocytes, and explore mechanisms of adipose-mediated therapy resistance.

Based on previous 3D co-culture studies of tumour cells, epithelial cells, and fibroblasts^26,60^, several approaches of culturing cells within a 3D matrix were tested and optimised. Following optimisation of culture conditions, including media type, duration (data not shown), collagen concentration (Fig. 1a, b) and cell densities (Fig. 1c), we demonstrated that by co-culturing the five above-mentioned key cell types of the obese breast TME in a collagen I matrix, we were able to recapitulate the inflamed adipose/tumour border observed in obese breast cancer patients.

As myoepithelial cells are tumour suppressors in normal breast and during early breast cancer development^61^, we assessed whether this cell type was required for an organotypic model of breast cancer. We observed that MCF-7 cells failed to form their typical ‘mass’ morphology in quadra-cultures when MECs were absent (Fig. S7). Therefore, it supports that MECs may play a role in the initial development of BC.

Our study proposes a simple approach to generating ‘obese’ adipocytes in vitro. Treatment of MA with palmitate in 2D culture resulted in hypertrophy and decreased mRNA expression levels of key mature adipocyte markers (Fig. 2). Generation of adipocytes in 3D spheroids formed physiologically relevant structures that displayed hypertrophy and metabolic dysregulation associated with obesity (Fig. 3).

This penta-culture system replicates the cell densities observed in ER^+^ breast cancer patient samples (Figs. 1c, 4e). Here, we show that cellular densities and localisation within an organotypic system are distinct in non-obese vs obese culture conditions (Fig. 4e and Fig. S4b). In a setting modelling obesity, we observed an increased density of fibroblasts distributed throughout the collagen gel, localisation of macrophages around tumour islands and a loss of myoepithelial cells, as compared to non-obese cultures. Increased fibroblast density has been previously observed in pre-clinical models of obesity-associated BC, hypothesised to be a result of adipocyte delipidation and de-differentiation^14,15^. Therefore, our model recapitulates obesity-associated inflammation and increased fibrosis, which may have potential implications for anti-cancer therapy response.

Previous studies suggest that obesity not only promotes breast cancer aggressiveness but is also associated with poor treatment outcome^12,62,63^. However, the underlying mechanisms of obesity-associated therapy resistance remain elusive. To investigate the utility of our organotypic system as a drug testing platform, we assessed the impact of three commonly used therapeutic agents and metformin on cell viability. We were particularly interested in metformin as it has demonstrated anti-tumorigenic properties both in pre-clinical and clinical studies, particularly in the context of obesity^64^. In pre-clinical models of endometrial cancer, metformin treatment was shown to reverse obesity-induced tumour aggressiveness via downregulation of lipid biosynthesis in obese compared to lean mice^53^. Notably, our results show that breast tumour cell monocultures showed therapeutic sensitivity to paclitaxel (Fig. 5a), which was lost upon co-culture with either MA or HA (Fig. 6a). Penta-cultures containing MA showed sensitivity to tamoxifen, doxorubicin and metformin treatment, as measured by loss of cell viability (Fig. 6c). Interestingly, in cultures treated with doxorubicin, Caspase-3-positive cells were mainly localised around adipocytes, which may indicate the presence of crown-like structures^65^.

Comparatively, in a setting modelling obesity, penta-cultures featured augmented sensitivity to metformin compared to non-obese cultures (Fig. 6d). Previous reports have shown that metformin treated BC cells display altered metabolic profiles^66–68^. Therefore, reduced ATP levels detected via cell viability assays may be due to a shift in metabolic pathway for generating ATP as opposed to an increase in apoptosis. Most notably, obese penta-cultures were completely resistant to paclitaxel treatment (Fig. 6c, d). This aligns with an earlier study, which observed paclitaxel resistance in MDA-MB-436 cells upon co-culture with adipocytes^12^. Prior research suggests that obesity-induced alterations in the TME enhance breast tumour proliferation^58^ and aggressive characteristics^69,70^. This may be due to a symbiotic relationship between BC cells and cancer-associated adipocytes, resulting in the production of proinflammatory cytokines^70^ and altered cellular metabolism^5^. Of note, we did not observe tamoxifen resistance in an obese setting, which has been previously reported in MCF-7 cells upon co-culture with mammary adipocytes^57^. However, a more recent study of an organotypic model of MCF-7 cells co-cultured with adipose-derived mesenchymal stem cells retained tamoxifen sensitivity^71^. This highlights the importance of cell culture conditions and the source of cell populations when modelling the obese breast TME in vitro.

Overall, our study provides evidence that obese adipose tissue in the breast TME can promote therapy resistance to commonly used chemotherapeutics such as paclitaxel and doxorubicin. Conversely, our data also suggest that metformin may provide therapeutic benefit in breast cancer patients with obesity. We are currently investigating a combination therapy approach to assess if metformin treatment can be used in combination with chemotherapies to restore sensitivity in vitro.

Although our study describes a 3D in vitro model that is both robust and reproducible, it does have some limitations. The use of cell lines, and the differentiation of adipose-derived stem cells into adipocytes, will likely result in distinct transcriptomic and metabolic differences to those that occur in vivo. Additionally, previous studies have shown the de-differentiation of adipocytes in collagen matrices^72^, and though not observed here, we do see increased collagen deposition around HA spheroids. However, using primary material as a source of adipocytes and other key populations can potentially circumvent this issue, hence future work, beyond the scope of this study will focus on the generation of an organotypic system comprised fully of primary-derived cells by culturing primary adipose tissue in a 3D matrix^59^ and incorporating primary breast tumour cells.

In summary, we have successfully generated a 3D in vitro model of obesity-associated breast cancer that incorporates five major cell types found in the breast in a physiologically relevant type I collagen matrix. The ability to produce adipose-like structures that form CLS in a relevant context validated the suitability of our model for investigating mechanisms of obesity-associated therapy resistance. Our system is robust, not prohibitively labour-intensive, and amenable to genetic manipulation. Furthermore, it offers the potential to dissect the mechanisms driving breast cancer progression and acquired therapy resistance in obesity.

## Methods

### Ethical approval

Experiments using primary-derived peripheral blood mononuclear cells (PBMCs) were conducted in accordance with a protocol approved by the University of Southampton Ethics Committee (ERGO: 19660.A7) and the East of Scotland Research Ethics Service, Tayside, UK Research ethical committee (REC), reference number: 16/ES/0048. Written consent was obtained, and ethical approval was issued for the BeGIN study by the University of Southampton Research Ethics Committee (REC) with ethics approval number: 14/EE/1297. Ethical approval was also gained for the use of primary-derived fibroblasts and myoepithelial cells from the Breast Cancer Now Tissue Bank (University of Southampton Ethics Committee; ERGO: 70449).

### Cell culture

Media, sera, and antibiotics were from Thermo Fisher Scientific. All cells were maintained in a humidified atmosphere of 10% CO_2_ at 37 °C unless otherwise stated. MCF-7 and T47D breast tumour cells were acquired from the ECACC. The normal fibroblast cell lines HFFF2 and MRC5 were kindly gifted from the Thomas and Blaydes Groups, respectively, at the University of Southampton. Primary-derived fibroblasts and myoepithelial cells (MECs) were obtained from the Breast Cancer Now Tissue Bank (https://breastcancernow.org/breast-cancer-research/breast-cancer-now-tissue-bank). Breast tumour cells and fibroblasts were cultured in DMEM (Gibco, 41965-039) supplemented with 10% foetal calf serum (FCS) (Sigma, f9665-500mML), 2 mM d-glucose sodium pyruvate (GP) (Gibco, 11360070), 30 mg/mL penicillin, 30 mg/mL streptomycin (P/S) (Gibco, 15140-122); referred to as 10% complete DMEM (cDMEM). The media was changed every 2–3 days. Cells were subcultured at 70–90% confluence. Myoepithelial cells were cultured in HAM F-12 medium (Gibco, 11765054) supplemented with 10% FBS, 2 mM GP, 30 mg/mL P/S, 0.5 µg/mL hydrocortisone (Sigma, H088-1G), 5 µg/mL insulin (Sigma, I9278-5ML), and 10 ng/mL human Epithelial Growth Factor (EGF) (Invitrogen, A42556). Cells were maintained at 5% CO_2_ at 37 °C, and media was changed every 3–4 days. Cells were subcultured at 70–80% confluence. Primary-derived MECs were cultured in HuMEC basal media (Gibco, 12752010) supplemented with the provided HuMEC supplement, 200 µl bovine pituitary extract (BPE), and 10 μg/mL Gentamicin (Gibco, 15710064). Key clinical information for each patient involved in fibroblast and myoepithelial cell derivation is listed below in Table 1.

**Table 1:** Clinical information for each patient from which primary fibroblasts or myoepithelial cells were derived

Sample type	Sample ID	BMI	Tumour type	Histology	Tumour grade
Fibroblasts	3225	30	IDC with squamous metaplasia	ER^+^/PR^+^/HER2^−^	3
Fibroblasts	2425	21	IDC	ER^+^/PR^+^/HER2^low^	2
MECs	1492	unknown	N/A	Normal	N/A

Samples were obtained from the Breast Cancer Now Tissue Bank. *IDC* invasive ductal carcinoma, *ER* oestrogen receptor, *PR* progesterone receptor.

#### Monocyte-derived macrophage (MDM) differentiation

PBMCs were isolated from leucocyte cones of healthy deidentified blood donor volunteers from the NHS Blood and Transplant bank. PBMCs were isolated following density gradient centrifugation using Lymphoprep (STEMCELL Technologies, 07801). Cells were then plated in 6-well plates (Corning) at a density of 2 × 10^7^ cells/plate in RPMI 1640 medium (Gibco, 31870-025) supplemented with 1% human AB serum (Sigma, H5667) and incubated at 37 °C for 2 h to allow monocytes to adhere to culture plates. Non-adherent cells were then removed with PBS washes. Twenty-four hours after plating, cells were treated with 100 ng/mL of macrophage colony-stimulating factor (MCSF) per well. Monocyte-to-macrophage differentiation was induced for 6–8 days in RPMI 1640 medium supplemented with 10% FBS, 2 mM GP, and 30 mg/mL P/S. Cells were refed on days 3 and 6 of culture.

#### Breast tumour 3D monocultures

Breast tumour cells were harvested, and the required volume was resuspended in ice-cold collagen type I (Corning, 354236). Collagen type I was diluted to the desired concentrations in PBS. Collagen-cell solutions were then seeded by pipetting 100 µl of solution into a 96-well plate and polymerised for 1 h. Growth media was then gently layered over the top of the collagen gel. Organotypics were cultured for 7 days, with media refreshed every two days.

#### Generating hypertrophic-like adipocytes in 3D

Adipose-derived stem cells (ADSCs) were acquired from Thermo Scientific. Cells were cultured in Mesenpro® media containing the provided supplement (Gibco, 12746012) as per the manufacturer’s instructions. The media was changed every 3–4 days. When ADSCs were ~80% confluent, cells were harvested by dissociation with TrypLE Express (Gibco, 12604013) and seeded at 7000 cells per well into ultra-low attachment 96-well plates (Corning, 10023683) in Mesenpro® media. Plates were then centrifuged at 200 × *g* for 10 min to promote spheroid formation. After 24 h, media was carefully removed from the wells and replaced with 100 µl of Stempro® differentiation medium (Gibco, A1007001) containing the provided supplement. Spheroids were maintained at 5% CO_2_ at 37 °C in Stempro® differentiation media for 21 days prior to the addition of palmitate. The media was refreshed every 2 days. After 21 days, mature adipocytes were fed with Stempro® differentiation media supplemented with 350 µM palmitate. Palmitic acid (Sigma, P0500-10G) was reconstituted in 99% ethanol, and a solution was prepared by adding 0.01 M fatty acid-free BSA (Sigma, A8806-1G). The selected concentration reflects the pathological levels of fatty acids (200–375 µM) adipose cells of obese individuals are exposed to^42^.

### Whole mount staining of 3D spheroid cultures

Three spheroids per culture condition were fixed in 4% paraformaldehyde (PFA) for 20–30 min at room temperature, washed three times with PBS and stored at 4 °C until staining. Prior to staining, spheroids were transferred to a 500 µl microcentrifuge tube (Eppendorf), with all subsequent steps carried out in the tube. Following PBS washes, spheroids were stained with DAPI (Thermo Scientific, 62248) at 1:500 dilution and BODIPY® 493/503 (Molecular Probes, 11540326) at 1:500 dilution. After 40 min incubation at RT, protected from light, spheroids were washed three times with PBS and stored at 4 °C until imaged. Fluorescence images were acquired with a confocal laser microscopy system (Leica TCS-SP8 Laser Scanning Confocal Microscope).

### Metabolic profiling of adipocytes

Mitochondrial respiratory function, glycolysis and fatty acid oxidation was assessed in MA and HA by means of a Seahorse XFe Extracellular Flux Analyser (Seahorse Bioscience, Agilent Technologies). Seahorse XFe Cell Mito Stress, Glycolysis Stress and Palmitate Oxidation Stress Test Kits were utilised according to the manufacturer’s instructions. ADSCs were seeded at a density of 10,000 cells per well of a 96-well seahorse plate, in 100 µl of culture media. After 24 h, the growth medium was replaced with differentiation medium and cultured until mature (21 days) or hypertrophic (32 days) as above. Oxygen consumption rate (OCR) and extracellular acidification rate (ECAR) were normalised to the protein content of cells using a Bradford assay.

### RNA extraction and qPCR

Adipocyte spheroids were collected in a 1.5 mL microcentrifuge tube. For RNA extraction, 700 µl of Qiazol Lysis Reagent (Qiagen, 79306) was added to each tube and incubated at RT for 5 min. Next, 140 µl of chloroform was added and mixed vigorously by shaking. After centrifugation at 12,000 × *g* for 15 min at 4 °C, the aqueous layer was removed and transferred to a new tube for RNA extraction. RNA was extracted using the miRNAeasy Kit (Qiagen, 217204) and eluted in 30 µl RNase-free water. The RNA quality and concentration was determined using Nanodrop. RNA was reverse transcribed into cDNA using SuperScript II Reverse Transcriptase (Invitrogen, 18064014). qPCR was performed using TaqMan Fast Advanced Master Mix, MicroAmp Fast Optical 96-well Reaction Plates (both Applied Biosystems) and TaqMan primers for human (h) PPARG (Hs01115513_m1), hFABP4 (Hs01086177_m1), hPLIN1 (Hs00160173_m1), hADIPOQ (Hs00977214_m1), hLEP (Hs00174877_m1) and hACTB (Hs99999903_m1). Data were analysed using the comparative CT method relative to hACTB.

### Glycerol release assays

Lipolytic activity of adipocytes was measured via glycerol release. Briefly, adipocyte spheroids were incubated in 100 µl RPMI for 3 h at 37 °C with or without 1 µmol/L isoprenaline (Sigma Aldrich, I6504-100MG) to evaluate induced and basal lipolysis, respectively. At the end of the incubation period, the supernatant was collected to quantify the amount of glycerol released using the Free Glycerol Reagent kit (Sigma Aldrich, F6428-40ML) according to the manufacturer’s instructions. Optical density was recorded on a VarioSkan Flash plate reader (Thermo Fisher Scientific) at 540 nm.

### 3D organotypic penta-cultures

Adipocyte spheroids were collected in microcentrifuge tubes and seeded at three spheroids per well into a 96-well plate. Spheroids were left to incubate at 37 °C whilst harvesting other cell populations and preparing collagen solutions. Breast tumour cells were directly co-cultured in a 1.0 mg/mL collagen type I matrix with myoepithelial cells, macrophages, and fibroblasts. Collagen was diluted with 10X DMEM low-glucose (Sigma, D2429-100ML), serum-free DMEM and neutralised with 1 M NaOH. Confluent monolayers of cells were harvested as above and mixed with ice-cold collagen solutions. Collagen-cell solutions were seeded by pipetting 100 µl of solution into a 96-well plate and polymerised for 1 h. Complete DMEM was then gently layered over the top of the collagen gel. Organotypics were cultured for 7 days, with media changes every 2 days.

#### Harvest of organotypic cultures for immunohistochemistry

Seven days after culture, organotypics were harvested. Supernatants were collected in 1.5 mL microcentrifuge tubes and stored at −80 °C. Wells were gently rinsed with 1X PBS and organotypics were transferred into a bijou containing 2 mL of 10% formalin solution, using a small sterile spatula. Organotypic samples were fixed in formalin for 1–2 days prior to immunohistochemistry.

#### Immunohistochemistry

Research Pathology (University Hospital Southampton) performed Haematoxylin and Eosin (H&E) and Ki67 staining on slides generated from paraffin-embedded blocks of organotypic samples. In summary, whole organotypic samples were fixed in formalin, processed using a Leica Peloris III tissue processor then embedded in paraffin and sectioned at 4 µm using a microtome, with the first section at the edge of the block and the second deeper section at the centre. Sections were then stained with H&E or Ki67.

Immunohistochemistry for Pan-cytokeratin (AE1/AE3), p63, α-SMA, CD68 and Perilipin-A as markers of breast tumour cells, myoepithelial cells, fibroblasts, macrophages, and adipocytes respectively, were performed on 4 µm sections of paraffin-embedded organotypic samples using BOND Rx Fully Automated Research Stainer (Leica Microsystems, U.K.), using BOND reagents according to the manufacturer’s instructions. Sections were deparaffinised, pre-treated for heat-induced Ag retrieval (BOND ER1/ER2 protocol), and incubated with hydrogen peroxide followed by primary antibody. Mouse or rabbit Abs were subsequently bound to the Poly-HRP IgG reagent before incubation with 3,3′-diaminobenzidine (DAB) (Leica, DS9800). For dual staining, the sections were subsequently incubated with another primary antibody, which was then bound to the Poly-HRP IgG reagent (Leica, 2MH-050). The substrate chromogen Refine Red (Leica Microsystems, DS9390) was applied, and sections were counterstained using haematoxylin. Slides were mounted using CV Ultra mounting media (Leica, 14070936261). Antibodies used were anti-cytokeratin (DAKO, 11515003), anti-p63 (Leica, 7JUL) at 1:25 dilution, anti-CD68 (DAKO, PG-M1) at 1:250 dilution, α-SMA (Abcam, 1A4) at 1:2000 dilution, anti-Perilipin-A (Abcam, ab3526) at 1:200 dilution, and anti-CD68 (DAKO, PG-M1) at a 1:100 dilution. Formalin-fixed and paraffin-embedded human breast tissue, human angiosarcoma, human spleen and human tonsil were used as positive controls.

### Image analysis

Images of multiplex IHC slides from full face sections of ER+ and TNBC breast cancer samples from the BeGIN study (Breast Cancer: Correlating Genetic, Immunological and Nutritional Predictors patient cohort; not published) were previously stained for pan-cytokeratin (AE1/AE3), α-SMA and CD68 (see Table 2 for key clinical characteristics). All immunostained slides were previously scanned using the Zeiss Imager.Z1 upright microscope equipped with the AxioCam MRc5 camera (Zeiss, Germany), using a predefined protocol at 20Χ magnification. These images were analysed using ImageJ (version 1.53) (http://imagej.nih.gov/ij/). The plugin Colour Deconvolution^73^ was used to quantify the percentage area of the section positively stained for pan-cytokeratin, α-SMA and CD68 in both ER+ and TNBC patients. The same plugin was also used to determine the number of cells positive for specific markers via IHC of organotypic samples. The Ki67 scores were defined as the percentage of positively stained cells among the total number of cancer cells. Organotypic samples stained for H&E were scored manually in ImageJ by counting the number of nuclei. The number of differentiated adipocytes and area of adipocytes stained for Oil Red O or BODIPY® 493/503, was quantified in ImageJ using the free-hand or elliptical measure tool.

**Table 2:** Key clinical parameters of each patient from the BeGIN study, in which tissue sections were stained with pan-cytokeratin,α-SMA and CD68

Patient ID	Sex	Age	BMI	Histology	Pathological stage	Histological tumour grade
SGH001	F	58	25.6	ER+/PR+/HER2−	T1N0	1
SGH005	F	59	30.1	ER+/PR+/HER2−	T1N0	2
SGH006	F	72	24.3	ER−/PR−/HER2−	T1N0	2
SGH007	F	57	30.8	ER+/PR+/HER2−	T1N0	2
SGH008	F	69	27.6	ER+/PR+/HER2−	T2N1	3
SGH010	F	48	30.5	ER+/PR+/HER2−	T2N0	3
SGH013	F	55	28.6	ER−/PR−/HER2−	T2N0	2
SGH014	F	55	36.1	ER+/PR+/HER2−	T1N0	2
SGH015	F	52	20.2	ER+/PR+/HER2−	T1N0	1
SGH016	F	68	28.6	ER+/PR+/HER2−	T2N1	2
SGH018	F	72	23.2	ER+/PR+/HER2−	T1N0	2
SGH021	F	69	29.3	ER−/PR−/HER2−	T2N0	1
SGH022	F	63	17.8	ER+/PR+/HER2−	T1N1	2
SGH023	F	58	35.8	ER−/PR−/HER2−	T1N0	2
SGH097	F	73	29.8	ER−/PR−/HER2−	T2N0	3
SGH101	F	40	25.2	ER−/PR−/HER2−	T2N1	3
SGH106	F	41	20.5	ER−/PR−/HER2−	T1N0	3
SGH108	F	66	38.3	ER−/PR−/HER2−	T1N0	3
SGH149	F	64	25.5	ER−/PR−/HER2−	T2N1	2
SGH158	F	47	50.8	ER−/PR−/HER2−	T1N0	3

### Drug testing

Paclitaxel was purchased from Thermo Scientific (10482875) and dissolved in ethanol. Tamoxifen was purchased from Sigma Aldrich (T5648-1G) and dissolved in ethanol. Ready-use doxorubicin was purchased from (Merk (D5220-1ML) and Metformin was purchased from Sigma Aldrich (D150959-5G), both were made up in complete DMEM.

#### CellTiter-Glo® 3D viability assays

To evaluate the different inhibitory effects of a panel of drugs on the cell viability of organotypic cultures, the CellTiter-Glo® 3D assay was performed according to the manufacturer’s instructions (Promega, G9681). On day 7 of culture, organotypic samples were treated with 10 µM Paclitaxel, 10 µM Tamoxifen, 50 µM Doxorubicin or 25 µM Metformin for 48 h. All drug solutions were made up to the required concentrations in complete DMEM. After 48 h, the supernatant was removed, and organotypic samples were transferred to clear-bottomed, white 96-well plates in 25 µl of growth medium per well. Then, 50 µl of CellTiter-Glo® 3D substrate was added at a 1:0.5 ratio to culture medium and mixed for 5 min using an orbital shaker. The plate was left to incubate for 25 min at room temperature. The luminescence was measured using a VarioSkan Flash plate reader instrument (Thermo Fisher Scientific).

### Statistical analyses

Statistical analyses were performed in GraphPad Prism (version 9.4.1). Paired *t*-tests were used to test for associations between the percentage of CK-positive cells, CD68-positive and α-SMA-positive cells in patient-matched multiplex slides. Unpaired t-tests were used to test for associations between the mRNA expression levels of adipogenic markers in MA and HA. One-way ANOVA testing for Dunnett’s multiple comparisons was used to compare drug treatments in penta-cultures containing MA or HA spheroids. For data without a normal distribution, Dunn’s multiple comparison test was used. Statistically significant ****P < 0.0001, ***P < 0.001, **P < 0.01, *P < 0.05 and non-significant (ns) *P* value ≥ 0.05.

## Supplementary information

Development of 3D obesity associated breast cancer model_supplementary figures_v3

## Data Availability

No large datasets were generated or analysed during the current study.

## Abbreviations

2D: Two-dimensional
3D: Three-dimensional
ADSC: Adipose-derived stem cell
ATMs: Adipose tissue macrophages
BC: Breast cancer
BMI: Body mass index
BME: Basement membrane extract
BPE: Bovine pituitary extract
BSA: Bovine serum albumin
CAFs: Cancer associated fibroblasts
CLS: Crown-like structures
CTR: Control
DAPI: 4′,6-diamidino-2-phenylindole
DCIS: Ductal carcinoma in situ
DMEM: Dulbecco’s modified Eagle’s medium
DOX: Doxorubicin
ECAR: Extracellular acidification rate
ECM: Extracellular matrix
ER: Oestrogen receptor
FCS: Foetal calf serum
FFPE: Formalin-fixed paraffin embedded
H&E: Haematoxylin and Eosin
HA: Hypertrophic adipocytes
HR: Hormone receptor
IF: Immunofluorescence
IL-6: Interleukin 6
IL-8: Interleukin 8
MA: Mature adipocytes
MECs: Myoepithelial cells
MET: Metformin
OCR: Oxygen consumption rate
OXPHOS: Oxidative phosphorylation
PBS: Phosphate-buffered saline
PCX: Paclitaxel
RT: Room temperature
TME: Tumour microenvironment
TMX: Tamoxifen
TN: Triple negative
TNF-α: Tumour necrosis factor alpha
