# Author Correction: Development and characterisation of a novel 3D in vitro model of obesity-associated breast cancer as a tool for drug testing

**DOI:** 10.1038/s41523-025-00823-x

**Published:** 2025-09-13

**Authors:** Rhianna R. R. Blyth, Stèphanie A. Laversin, Russell B. Foxall, Constantinos Savva, Ellen Copson, Ramsey I. Cutress, Charles N. Birts, Stephen A. Beers

**Affiliations:** 1 Antibody and Vaccine Group, Centre for Cancer Immunology, School of Cancer Sciences, Faculty of Medicine, University of Southampton, Southampton, SO16 6YD UK; 2 School of Cancer Sciences, Faculty of Medicine, University of Southampton, Southampton, SO16 6YD UK; 3 NIHR Southampton Biomedical Research Centre, University Hospital Southampton NHS Foundation Trust, Southampton, SO16 6YD UK; 4 School of Biological Sciences, Faculty of Environmental and Life Sciences, University of Southampton, Southampton, SO17 1BJ UK; 5 Institute for Life Sciences, University of Southampton, Southampton, SO17 1BJ UK

**Keywords:** Breast cancer, Cancer metabolism, Cancer microenvironment, Cancer models

Correction to: *npj Breast Cancer* 10.1038/s41523-025-00766-3, published online 30 May 2025

In this article, an incorrect version of Figure 5 was inadvertently uploaded after acceptance. The original article has been corrected.

