# Supplementary material for: Development and characterisation of a novel 3D in vitro model of obesity-associated breast cancer as a tool for drug testing
Source: NPJ Breast Cancer. 2025 May 30;11:50. doi: 10.1038/s41523-025-00766-3 (PMC12125370; doi:10.1038/s41523-025-00766-3)
Supplement: Supplementary file 1 — Development of 3D obesity associated breast cancer model_supplementary figures_v3 [file 41523_2025_766_MOESM1_ESM.pdf]

**Development and characterisation of a novel 3D in vitro model of obesity associated breast cancer as a tool for drug testing**

**Supplementary figures**

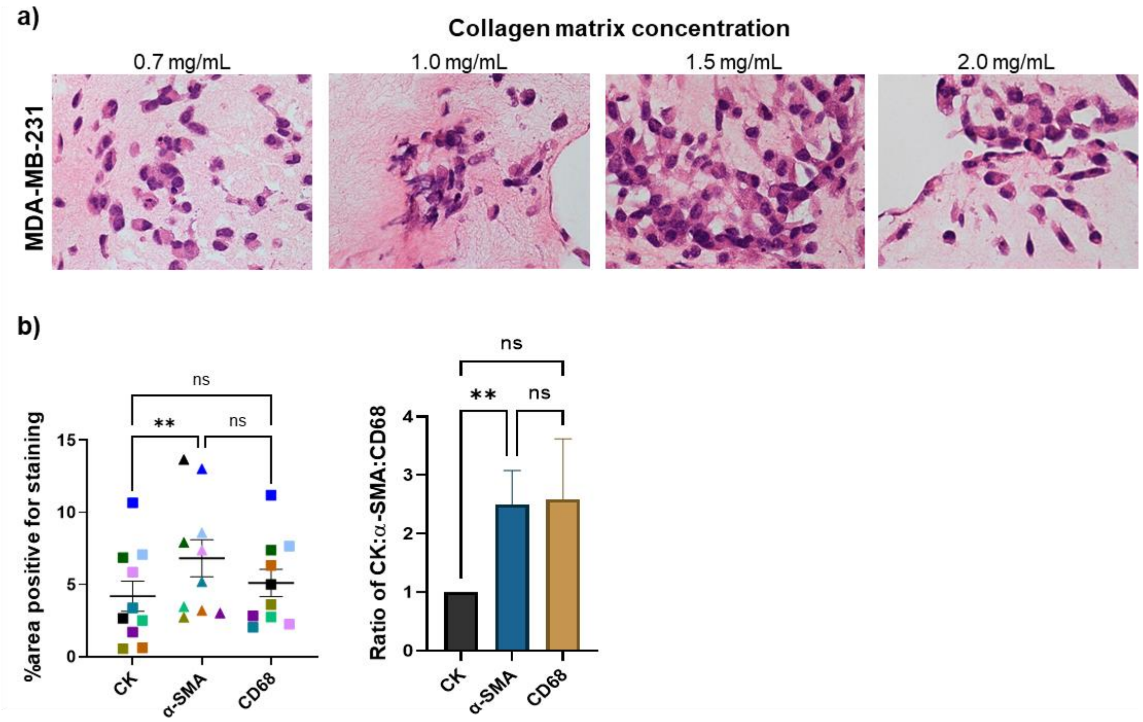


**Figure S1. Triple-negative breast cancer cells require a stiffer collagen matrix in3D culture.** (a) H&E staining of organotypic MDA-MB-231 monocultures in collagen type I matrices ranging in concentration from 0.7-2.0 mg/mL for 7 days. H&E stained 4 µm sections of Formalin-fixed Paraffin-embedded (FFPE) organotypic cultures are shown. Each brightfield image is obtained from a single organotypic model at 40x magnification, scale bar: 20 µm. (b) Percentage positive staining of pan-cytokeratin (filled squares), α-SMA (filled triangles) and CD68 (filled squares) in 10 TNBC patient punch biopsy samples, colour coded according to patient ID. Ratio of fibroblasts to breast cancer cells. Percentage positive staining quantified in ImageJ using the Colour Deconvolution plugin. One-way ANOVA with Friedman test used to test for statistical significance; *P<0.05, **P<0.01, and non-significant (ns) P>0.05.

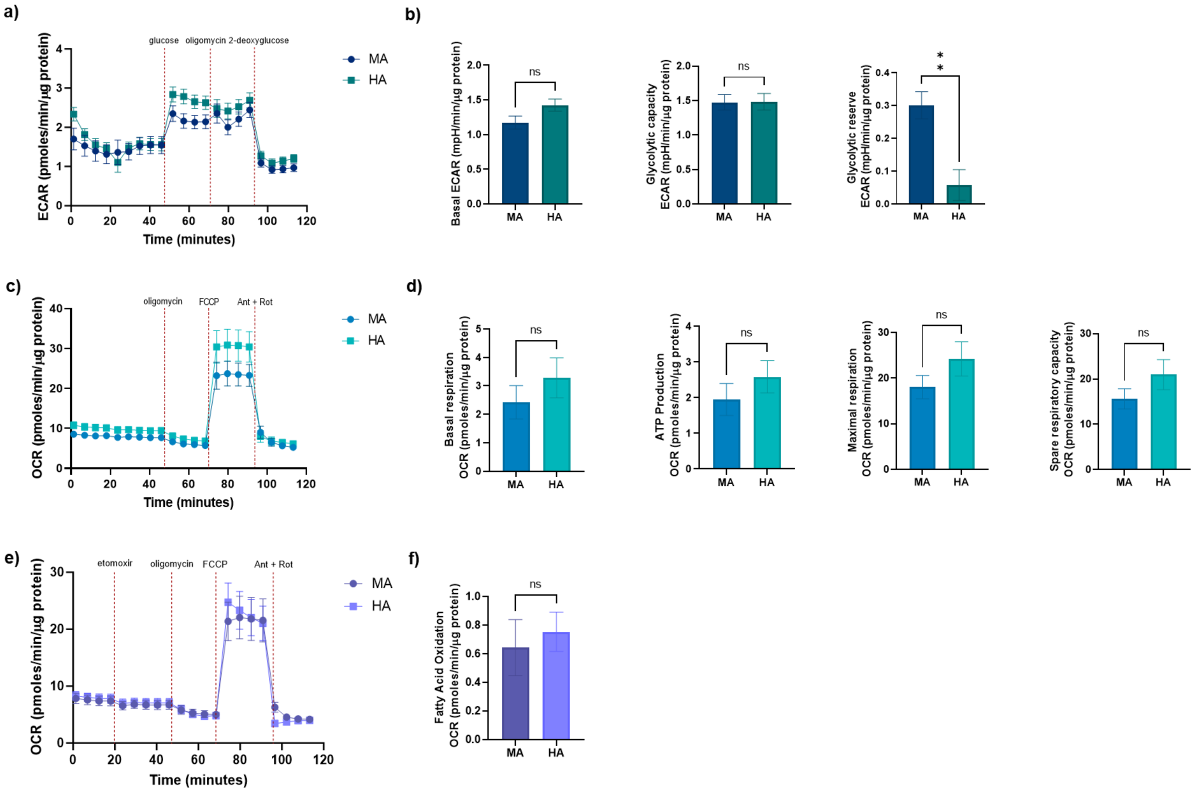


**Figure S2. Comparative bioenergetic profiles of mature and hypertrophic-like adipocytes.** (a) Graphical representation of extracellular acidification rates (ECAR) of mature (dark blue) and hypertrophic-like adipocytes (dark green). (b) Glycolytic capacity, basal glycolysis, and glycolytic reserves of mature and hypertrophic-like adipocytes. Cells were assayed in DMEM supplemented with 2 mM glutamine, with sequential injection of 5 mM glucose (port B), 1 µM oligomycin (port C), and 50 mM 2-deoxyglucose (port D). (c) Oxygen consumption rates (OCR) of mature adipocytes (blue) and hypertrophic-like adipocytes (turquoise). (d) Basal respiration, ATP production, maximal respiration, and spare respiratory capacity. Cells were assayed in DMEM supplemented with 2 mM glutamine, with sequential injection of 1 µM oligomycin (port B), 0.5 µM FCCP (port C) and 0.5 µM each of antimycin/rotenone (port D). Total ATP production rates shows the contribution of oxidative phosphorylation in mature and hypertrophic-like adipocytes after addition of glucose and then oligomycin. (e) Oxygen consumption rates (OCR) of mature adipocytes (dark purple) and hypertrophic-like adipocytes (lilac). (f) Total long-chain fatty acid oxidation in mature and hypertrophic-like adipocytes after addition of etomoxir. Mature differentiated adipocytes measured after 21 days in culture. Hypertrophic-like adipocytes differentiated for 32 days in differentiation medium. All data are means ± SEM. Statistical significance between groups was assessed using unpaired t-tests, **P<0.01, ns: non-significant P>0.05.

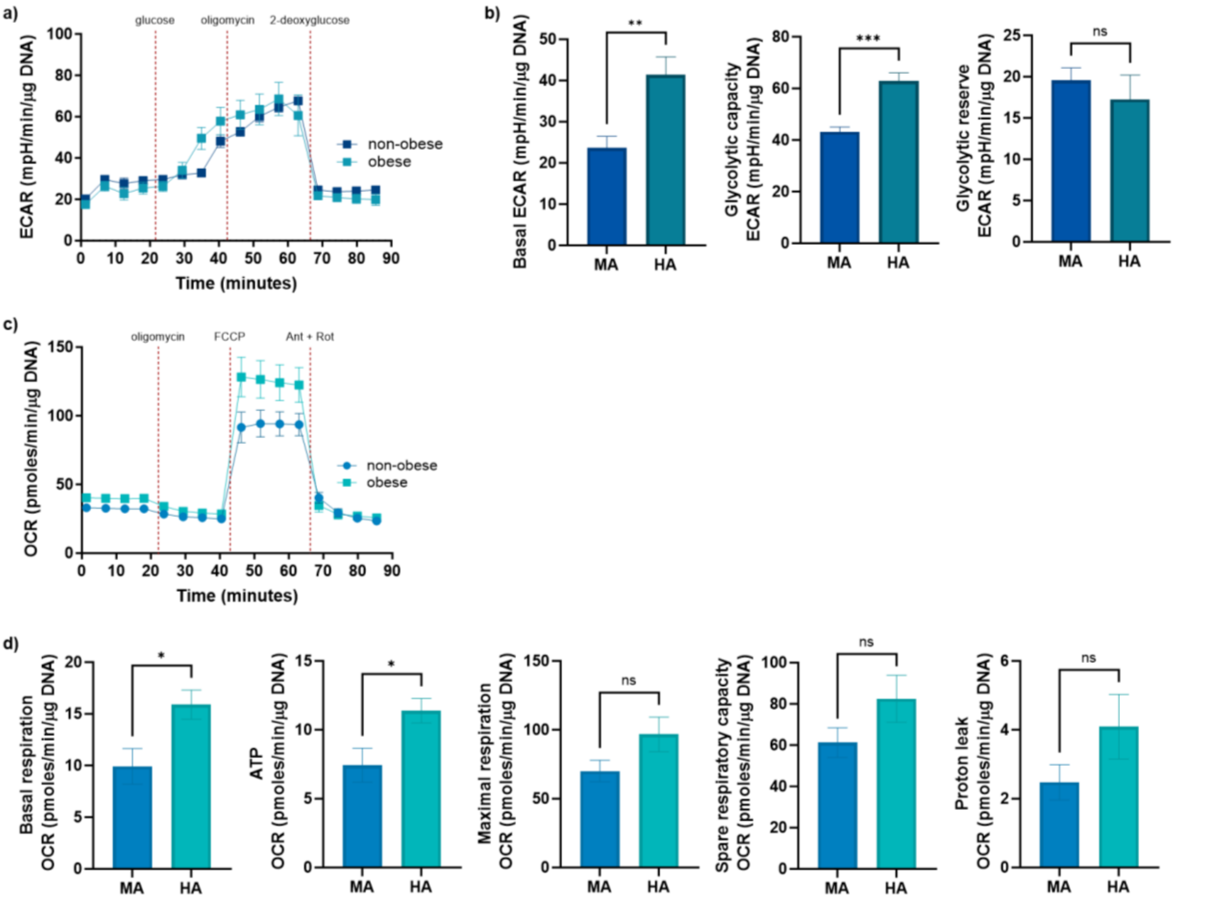


**Figure S3. Bioenergetic profiles of adipocytes spheroids.** (a) Extracellular acidification rates (ECAR) of mature adipocytes (dark blue) and hypertrophic-like adipocytes (dark green). (b) Glycolytic capacity, basal glycolysis and glycolytic reserves of mature (MA) and hypertrophic (HA) adipocyte spheroids. Cells were assayed in DMEM supplemented with 2 mM glutamine, with sequential injection of 5 mM glucose (port B), 1 µM oligomycin (port C), and 50 mM 2-deoxyglucose (port D). (c) Oxygen consumption rate (OCR) over time. (d) Basal respiration, mitochondrial ATP production, maximal respiration, spare respiratory capacity and proton leakage. All data are means ± SEM of 4 wells across three plates (n=3 independent Seahorse run). Unpaired t-test used to test for statistical significance ***P<0.001, **P<0.01, *P<0.05 and non-significant (ns) P>0.05.

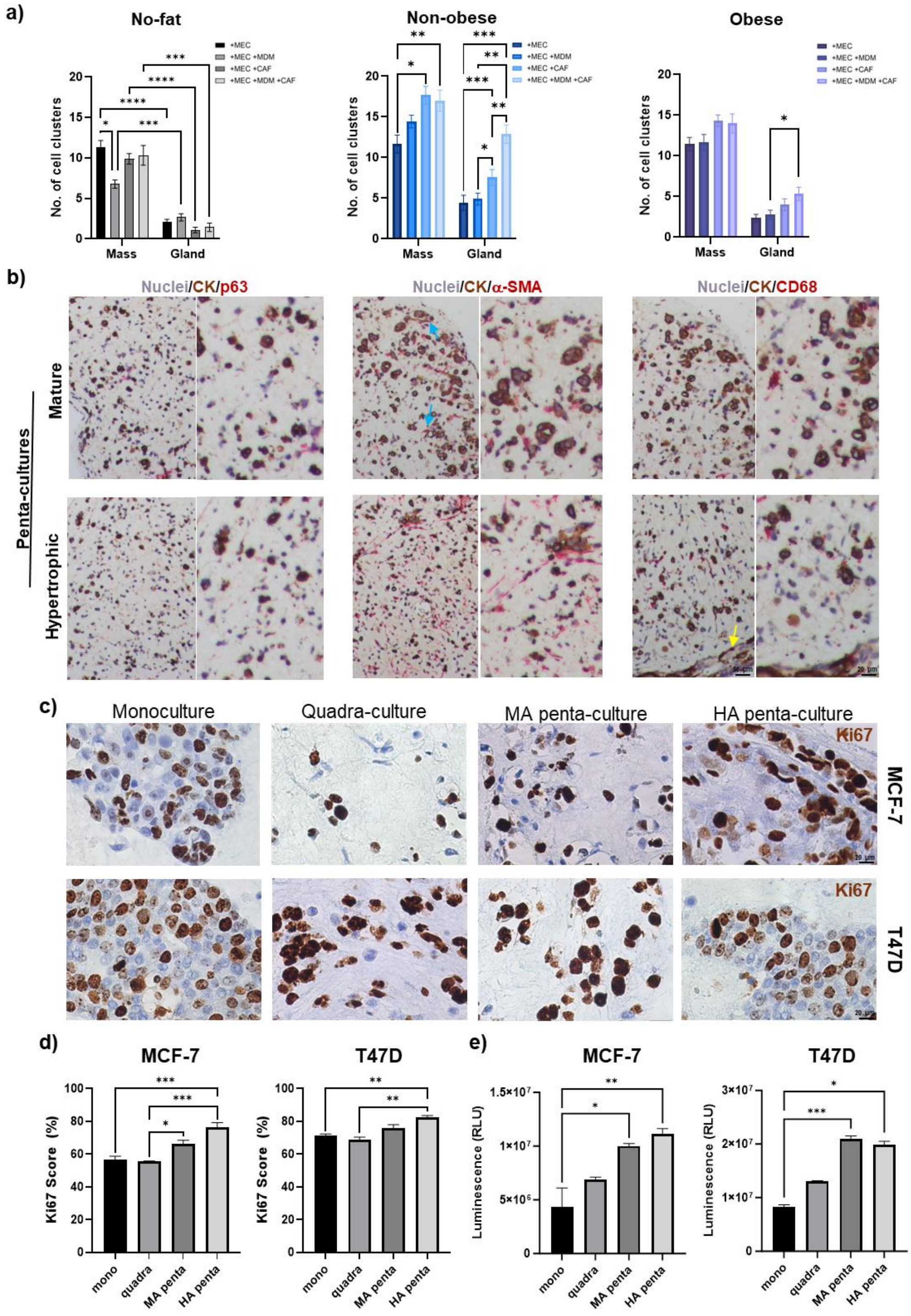


**Figure S4. ER+ breast tumour cells have enhanced proliferation in 3D penta-cultures.** (a) Quantification of morphologies of MCF-7 organotypics in different co-culture conditions (no-fat quadra-culture, non-obese or obese penta-cultures containing mature or hypertrophic adipocytes respectively. (b) Organotypic sections stained for pan-cytokeratin (CK- brown) p63 (Refine red), α-SMA (Refine red) and CD68 (Refine red). Haematoxylin was used to counter stain nuclei. Representative brightfield images, scale bar= 50 µm or 20 µm as indicated. (c) Ki67 staining of organotypic samples of MCF-7 or T47D cells cultured alone (monoculture), with myoepithelial cells, macrophages and fibroblasts (quadra-culture) or with the addition of mature (MA) or hypertrophic (HA) as penta-cultures. (d) Quantification of Ki67 staining, as a percentage of total number of cells. (e) Luminescence values determined via CellTiter-Glo® 3D assay, according to manufacturer's instructions, in breast tumour organotypics. Data represents mean ± SEM from three independent experiments.

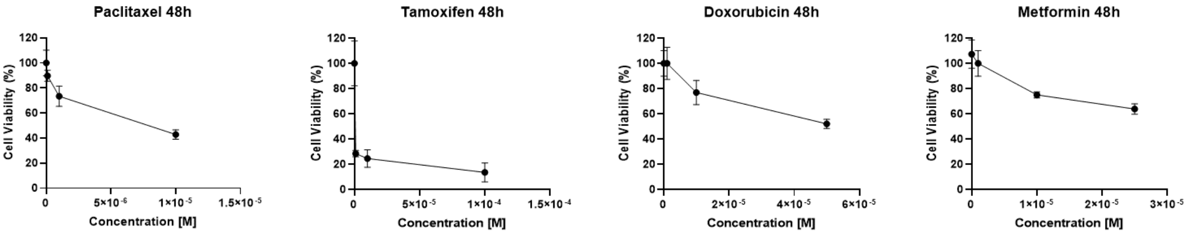


**Figure S5. ER⁺ breast tumour cells are sensitive to paclitaxel and tamoxifen when cultured in 3D.** Cell viability was determined with a CellTiter-Glo® 3D assay, according to manufacturer's instructions, in breast tumour organotypic monocultures. These cultures were treated for 48 hours with three doses of one of four therapies: 0.1 µM, 1 µM, 10 µM paclitaxel, 1 µM, 10 µM, 100 µM tamoxifen, 1 µM, 25 µM, 50 µM doxorubicin, 1 µM, 10 µM, 25 µM metformin, or a DMSO control. Luminescence values were converted into cell viability by normalising drug-treated cultures to the control cultures. Data was obtained from three independent experiments.

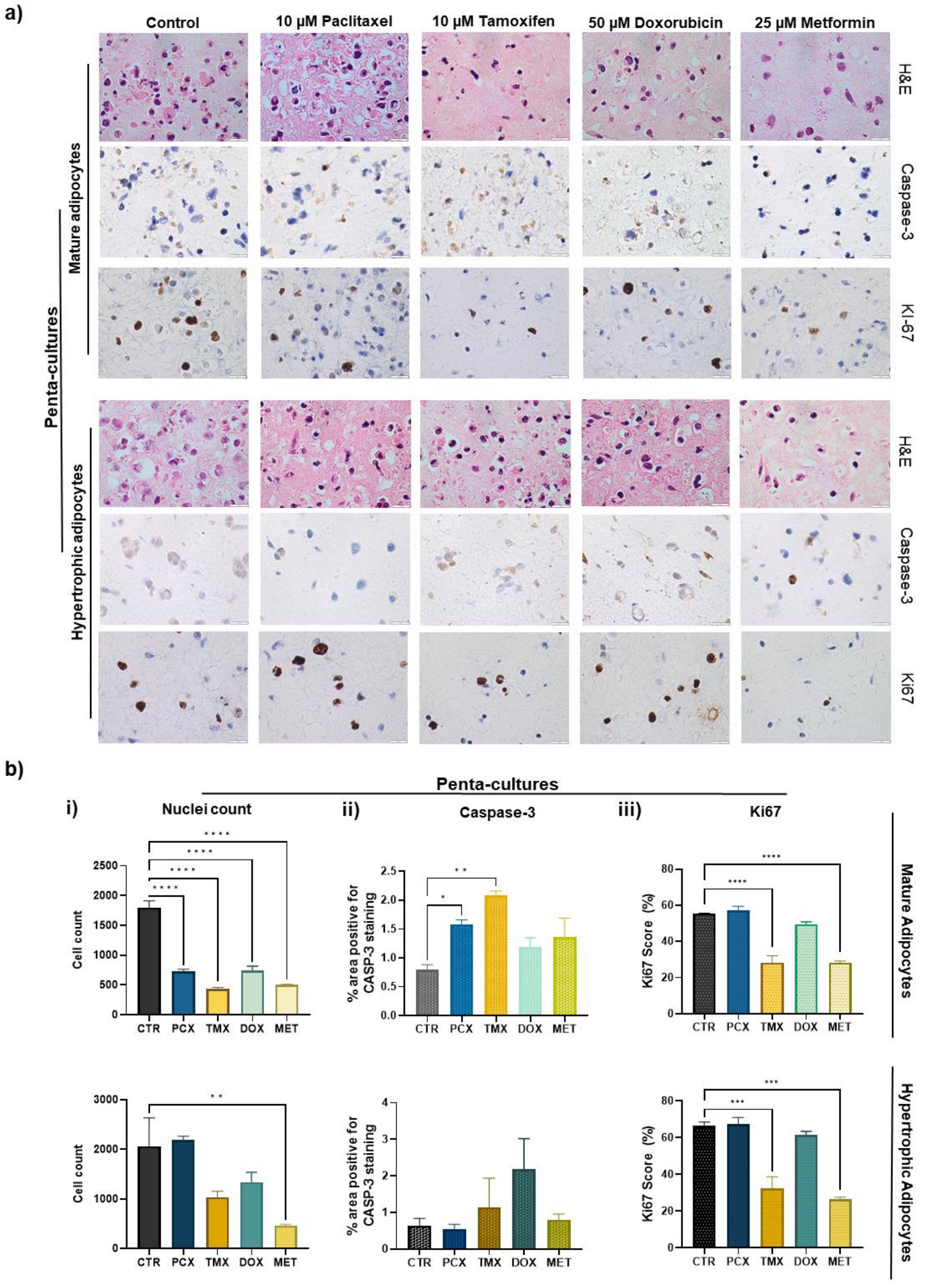


**Figure S6. Breast tumour cells are resistant to paclitaxel but sensitive to metformin in an obese setting.** Organotypic cultures were treated for 48 hours with one of four therapeutic compounds: 10 µM paclitaxel (PCX), 10 µM tamoxifen (TMX), 50 µM doxorubicin (DOX), 25 µM metformin (MET), or a DMSO control (CTR). (a) FFPE blocks generated from treated and untreated organotypic samples were sectioned, stained for H&E, Caspase-3 or Ki67. Representative brightfield images at 40x magnification are shown, scale bar= 20 µm. (bi) Number of nuclei manually counted in ImageJ. (bii) Sections stained for Caspase-3 or (biii) Ki67 were analysed in ImageJ for percentage of area positive for staining using the Color Deconvolution plugin. Statistical significance between groups was assessed using an ordinary One-Way ANOVA with Dunnett's or Kruskal-Wallis comparison test. ****P<0.0001, ***P<0.001, **P<0.01, *P<0.05. All other comparisons were non-significant (P>0.05).

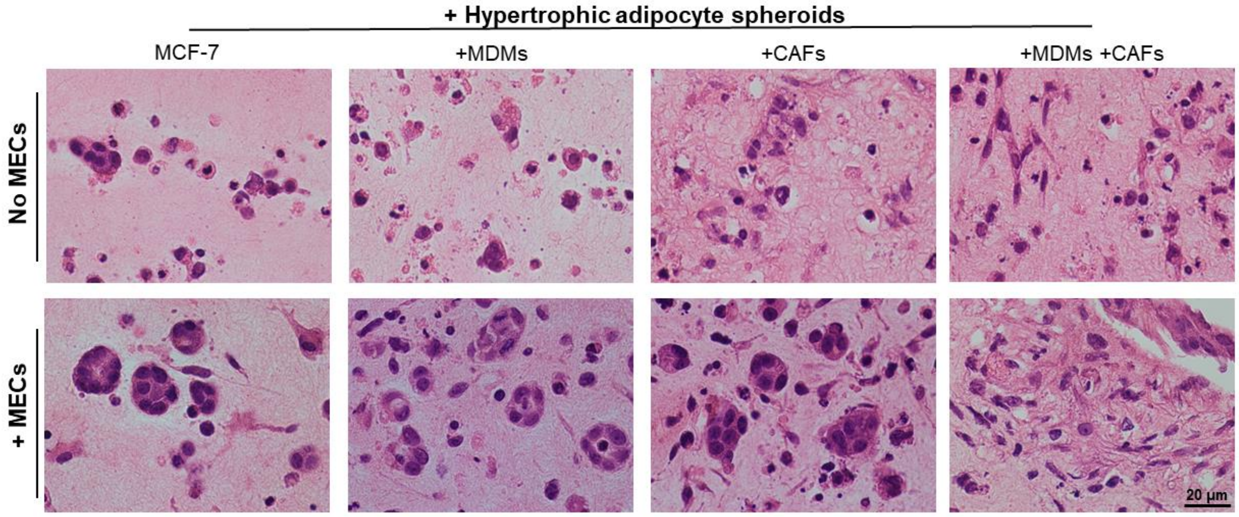


**Figure S7. Myoepithelial cells are required for MCF-7 cell growth in an organotypic model of BC.** (a) H&E staining of organotypic co-culture samples. MCF-7 breast tumour cells co-cultured with monocyte-derived macrophages (MDMs), cancer-associated fibroblasts (CAFs), and hypertrophic adipocyte spheroids in a collagen type I matrix for 7 days in either the presence or absence of myoepithelial cells (MECs). Representative images taken at 40x magnification under brightfield. Scale bar= 20 µm.
